# Non-Equilibrium Wigner Function and Application to Model of Catalyzed Polymerization

**DOI:** 10.3390/e26020104

**Published:** 2024-01-24

**Authors:** Ramon F. Alvarez-Estrada

**Affiliations:** Departamento de Física Teórica, Universidad Complutense de Madrid, 28040 Madrid, Spain; rfa@ucm.es

**Keywords:** non-equilibrium Wigner function and hierarchy for moments, short thermal wavelength and long-time regimes, approximate Smoluchovski equation, catalyzed polymerization

## Abstract

The quantum Wigner function and non-equilibrium equation for a microscopic particle in one spatial dimension (1D) subject to a potential and a heat bath at thermal equilibrium are considered by non-trivially extending a previous analysis. The non-equilibrium equation yields a general hierarchy for suitable non-equilibrium moments. A new non-trivial solution of the hierarchy combining the continued fractions and infinite series thereof is obtained and analyzed. In a short thermal wavelength regime (keeping quantum features adequate for chemical reactions), the hierarchy is approximated by a three-term one. For long times, in turn, the three-term hierarchy is replaced by a Smoluchovski equation. By extending that 1D analysis, a new model of the growth (polymerization) of a molecular chain (template or te) by binding an individual unit (an atom) and activation by a catalyst is developed in three spatial dimensions (3D). The atom, te, and catalyst move randomly as solutions in a fluid at rest in thermal equilibrium. Classical statistical mechanics describe the te and catalyst approximately. Atoms and bindings are treated quantum-mechanically. A mixed non-equilibrium quantum–classical Wigner–Liouville function and dynamical equations for the atom and for the te and catalyst, respectively, are employed. By integrating over the degrees of freedom of te and with the catalyst assumed to be near equilibrium, an approximate Smoluchowski equation is obtained for the unit. The mean first passage time (MFPT) for the atom to become bound to the te, facilitated by the catalyst, is considered. The resulting MFPT is consistent with the Arrhenius formula for rate constants in chemical reactions.

## 1. Introduction

Non-equilibrium quantum statistical mechanics has its own scientific importance [1,2,3,4,5,6,7,8,9,10,11,12,13,14,15,16,17], and its applications make it even more important. Part of the latter, of paramount relevance, are chemical reactions [18,19,20,21,22,23]: in them, typically, atoms/molecules in an initial state are not the same as those in the final one, while on the other hand, those processes occur in the presence or inside a statistical medium (for instance, a fluid). Here, chemical reactions can be understood in a broad sense and include, namely, biochemical processes [23]. The very formation and breaking up of bound states with discretized binding energies in chemical reactions unavoidably requires quantum mechanics and statistical mechanical concepts, even if other features in those processes can be accounted for in terms of classical physics for that purpose. For further knowledge regarding thermodynamics and statistical mechanics, see, for instance [24,25,26,27,28].

Before proceeding further, it seems adequate to remind the reader of part of the previous work that has motivated the present one. For that purpose, see [29,30] and references therein.

The non-equilibrium dynamics of a closed classical gas composed of a large number of identical non-relativistic particles was described by the classical Liouville distribution *f* and equation, which depend on the time *t* and on the positions and momenta of all particles with some suitable initial condition at t=0. It was assumed that only the inner part of the gas was off-equilibrium while the remainder of it was at equilibrium at absolute temperature *T*. The standard Boltzmann equilibrium distribution $f_{eq}$ with temperature *T*, being Gaussian in all momenta, generated a family of orthogonal polynomials in the latter: the standard Hermite polynomials. The latter, upon integrating on the momenta, enabled the introduction of non-equilibrium moments (depending on *t* and particle positions). The Liouville equation gave rise to an infinite three-term hierarchy for those classical moments. The hierarchy was entirely different from the standard Bogoliubov–Born–Green–Kirkwood–Yvon (BBGKY) hierarchy [1,3,4]. The hierarchy was solved in terms of suitable infinite continued fractions of operators. Those continued fractions suggested and allowed for the implementation of a long-time approximation. After further approximations, the lowest non-equilibrium moment was shown to satisfy a Smoluchovsky equation, which is formally similar to the one characterizing the so-called Rouse model in polymer dynamics [31]. An approximate approach to thermal equilibrium over a long time can then be established.

Attempts at extensions of those procedures to quantum processes faced two difficulties. First, it was not warranted that, contrary to the classical situation, quantum–mechanical distributions be non-negative in certain limited spatial domains. This, in turn, would, in general, prevent a direct use of the corresponding equilibrium distribution to generate, in the standard way, a family of orthogonal polynomials in momenta and, so, non-equilibrium moments. Such difficulty was bypassed by invoking a suitable generalization of the theory of orthogonal polynomials, as will be illustrated later in Section 2.3 of this work. The second difficulty was (and still continues partly to be) that, after having implemented that solution of the first difficulty, the resulting hierarchy for the non-equilibrium moments is not, in general, a three-term one: this demands proper analysis.

The present work presents: (1) a new study of non-equilibrium quantum statistical mechanics aimed at providing a new (at least, partial) solution to the second difficulty (Section 2) and (2) one possible application to a certain chemical reaction: namely, the one playing a key role in the polymerization of a molecular chain [32] (Section 3 and Section 4). In so doing, previous analysis will be generalized [30,33] in a non-trivial way.

Section 2 presents: a one-dimensional (1D) non-equilibrium quantum Wigner function (*W*) and dynamical equation, the introduction of a family of orthogonal polynomials generated by the equilibrium Wigner distribution ($W_{eq}$), a general n-term recurrence relation for non-equilibrium moments of the corresponding *W* (determined, in turn, by those orthogonal polynomials), a formal solution by combining continued operator fractions and series expansions thereof, an approximate three-term recurrence relation for short thermal wavelengths (still in the quantum regime), long-time approximations, and an irreversible Smoluchowski equation.

Section 3 treats a three-dimensional (3D) model for the addition (polymerization) of one single atom to a freely jointed molecular chain acting as a template (te) activated by a catalyst in a fluid at equilibrium at a given temperature.

Section 4 studies the model in Section 3 through 3D non-equilibrium quantum–classical Wigner–Liouville functions, a dynamical equation for the individual atom, the te, and the catalyst, and successive approximations (assuming that the te and catalyst are at thermal equilibrium) so as to yield a standard 3D non-equilibrium Wigner equation. At this stage, the 1D developments in Section 2 (short thermal wavelength and long-time approximations in Section 2) are extended to the above standard 3D equation, thereby yielding a Smoluchowski equation for the single atom, and a mean first passage time (MFPT) equation for the atom to become attached to the te chain.

Successive approximations are made for conditions suitable for chemical reactions.

Section 5 offers the conclusions and some discussions.

This work is a contribution to “180th Anniversary of Ludwig Boltzmann”, a Special Issue of Entropy.

## 2. One-Dimensional Wigner Function and Equation

### 2.1. General Aspects

We consider a simplified model of a microscopic non-relativistic particle of mass *m* in one spatial dimension (*x*) in a finite interval Ω (=(−L/2,+L/2)), which is large on the microscopic scale although possibly small on the macroscopic one, by omitting unnecessary details. The particle is subject to a real (time-independent and velocity-independent) potential V=V(x) in −L/2<x<L/2 and to a heat bath (HB) at absolute temperature *T*. The quantum particle Hamiltonian is $H=-\left(\hbar^{2}/2m\right)\left(\partial^{2}/\partial x^{2}\right)+V$, with *ℏ* being Planck’s constant. For definiteness, the wave functions may be assumed to fulfill Dirichlet boundary conditions in ∣x∣=L/2.

The conditions on the potential V=V(x) are:

V(x)=V(−x) (for simplicity, although strictly unnecessary).
V(x) is attractive (<0) in the interval −a<x<a (0<a<L/2); it is very small (with an arbitrary sign) in a<∣x∣<L/2 and vanishes fast as ∣x∣→L/2. Specifically, V(x) is finite everywhere, and its magnitude |V| is appreciable only in the limited interval −a<x<a; *a* is understood to be the range of *V*.
V(x) and all $d^{n}V\left(x\right)/dx^{n}$ for n=1,2,3,… are continuous everywhere.

In general, *H* has both a denumerably infinite number of discrete states with non-negative energy (an almost continuous spectrum: *L* being large but finite at the microscopic scale) and, in principle, a finite discrete spectrum (j=d) with energies $E_{d}<0$. With *j* being a general label, let $\varphi_{j}=\varphi_{j}\left(x\right)$ generically denote a suitably normalized eigenfunction of *H* with corresponding eigenvalue $E_{j}$.

As a simplifying assumption, V(x) does give rise to only one bound state (bound spectrum).

The denumerably infinite discrete (almost continuous) spectrum $E_{j}$ of *H* has a small spacing, approaches a continuous spectrum more the larger *L* is, and would become a continuous one (sweeping the continuous positive real axis $0<E_{j}<+\infty$) if $L^{-1}\to 0$. We shall always denote it by CS, even if the small $L^{-1}$ remains >0. The eigenfunctions corresponding to CS are $\varphi_{j}=\varphi_{k}\left(x\right)$, with j≡k being an almost continuous wavevector and eigenvalues $E_{j}=E_{k}=\hbar^{2}k^{2}/\left(2m\right)\geq 0$. The CS eigenfunctions are normalized through: $\left(\varphi_{k},\varphi_{k^{\prime }}\right)=\int dx\varphi_{k}^{*}\varphi_{k^{\prime }}=\delta_{k,k^{\prime }}$ (a Kronecker delta). Also, by taking into account the bound state: $\left(\varphi_{d},\varphi_{d}\right)=\int dx\varphi_{d}^{*}\varphi_{d}=1$ (normalized) and $\left(\varphi_{d},\varphi_{k}\right)=\int dx\varphi_{d}^{*}\varphi_{k}=0$. Integrations are performed over Ω. Hence, $\varphi_{d}$ and all CS $\varphi_{k}$ span two separate Hilbert subspaces $H_{d}$ and $H_{\mathrm{CS}}$. See [34,35,36].

The time (*t*) evolution of the quantum particle is given by the general density operator ρ=ρ(t) (a statistical mixture of quantum states) for t>0. It fulfills the (*t*-reversible) operator equation $\partial \rho /\partial t={\left(i\hbar \right)}^{-1}\left[H,\rho \right]$ ([H,ρ]=Hρ−ρH being the commutator) with initial condition $\rho \left(t=0\right)=\rho_{\mathrm{in}}$. The Hermitian and positive-definite linear operators ρ(t) and $\rho_{\mathrm{in}}$ act on the Hilbert space spanned by the set of all eigenfunctions of *H*. See [34].

Let $\beta ={\left(k_{B}T\right)}^{-1}$, with $k_{B}$ being Boltzmann’s constant. We shall introduce the fixed and physically relevant *x*-independent momentum and thermal wavelength:

(1) $$\begin{array}{ccc} & & q_{\mathrm{eq}}={\left(2m/\beta \right)}^{1/2},\lambda_{th}=\hbar /q_{\mathrm{eq}} \end{array}$$

We now resort to the non-equilibrium (reversible) Wigner function [1,2,4,6,7,8,37] at *t*, which reads formally:

(2) $$\begin{array}{ccc} & & W\left(x,q;t\right)=\frac{1}{\pi \hbar }\int dx^{\prime }\exp\left[\frac{2i}{\hbar }x^{\prime }q\right]\langle x-x^{\prime }|\rho \left(t\right)|x+x^{\prime }\rangle , \end{array}$$

The initial Wigner function $W_{\mathrm{in}}$ is given by Equation (2) by using ρ(t=0). The equilibrium density operator $\rho_{\mathrm{eq}}=\exp\left[-\beta H\right]$ determines the equilibrium Wigner function $W_{\mathrm{eq}}\left(x,q\right)$ formally as:

(3) $$\begin{array}{ccc} & & W_{\mathrm{eq}}\left(x,q\right)=\frac{1}{\pi \hbar }\int dx^{\prime }\exp\left(\frac{i2qx^{\prime }}{\hbar }\right)\langle x-x^{\prime }|\rho_{\mathrm{eq}}|x+x^{\prime }\rangle . \end{array}$$

A more specific analysis is required since Ω is not strictly an infinite interval. However, as *L* is large, we shall approximate spatial integrals as those for an infinite length interval when such approximations are harmless unless some specific discussion is required. Strictly speaking, as Ω is not an infinite interval, the $q^{\prime }$ are discretized momenta and $\int dq^{\prime }$ (which will appear later) should be interpreted as a series. However, as *L* is large, we shall disregard the small spacings in $q^{\prime }$ and understand $\int dq^{\prime }$, as the notation indicates, as an integral ($q^{\prime }$ thus varying continuously) instead of as a sum. This remark and interpretation will apply and be understood whenever integrations over momenta occur later. We shall accept that all integrals (or all series) over momenta converge for large values of the latter: explicit expressions and computations will support this assumption. Summarizing: as *L* is large and unless otherwise stated, we shall approximate using practical calculations (without writing it explicitly) spatial integrals by those in −∞<L<+∞ and series over momenta by integrations over them in −∞<q<+∞.

Integrability properties of the Wigner function hold and will not be considered here for brevity: they have been treated previously in [29,30] and, in particular, in references therein.

In principle, $\sum_{j}$ will denote sums over all eigenfunctions in j=d and j=CS; that is, the former includes the contribution of both the single (bound state) discrete eigenfunction plus that of an infinite summation over the whole CS ones. Since *L* is large, we approximate for the CS: $\sum_{j}\to \left(L/\left(2\pi \right)\right)\int dk$ as $L^{-1}\to 0$. Therefore, with the latter understanding for a large-interval Ω, $W_{\mathrm{eq}}$ is approximated as:

(4) $$\begin{array}{ccc} W_{\mathrm{eq}}\left(x,q\right)\;\; & =\;\; & \frac{1}{\left(\pi \hbar \right)}\int dx^{\prime }\exp\left(\frac{i2qx}{\hbar }\right)\sum_{j}\exp\left[-\beta E_{j}\right]\varphi_{j}\left(x-x^{\prime }\right)\varphi_{j}^{*}\left(x+x^{\prime }\right). \end{array}$$

While at T=0 there is no transition between the bound state and the CS ones; such a transition is indeed possible for T>0 (due to the HB) and can play a key role as $k_{B}T$ approaches $|E_{d}|$. We emphasize that the contribution of the bound state becomes more negligible the higher the temperature is (quasi-classical regime) and even disappears in the full classical regime. On the other hand, there seems to be no compelling reason for not using the quasi-classical or even classical formula as rough or zeroth-order approximations in regions where the contributions due to the bound states are negligible.

For t>0, the exact (*t*-reversible) dissipationless quantum master equation for the general off-equilibrium Wigner function *W* [6,37] is:

(5) $$\begin{array}{ccc} \frac{\partial W(x,q;t)}{\partial t}\;\; & = & \;\;-\frac{q}{m}\frac{\partial W(x,q;t)}{\partial x}+M_{Q}W, \end{array}$$

(6) $$\begin{array}{ccc} M_{Q}W\;\; & = & \;\;\int dq^{\prime }W\left(x,q^{\prime };t\right)\int \frac{idx^{\prime }}{\pi \hbar^{2}}\left[V\left(x+x^{\prime }\right)-V\left(x-x^{\prime }\right)\right]\exp\left[\frac{i2\left(q-q^{\prime }\right)x^{\prime }}{\hbar }\right]\\ \;\; & = & \;\;\frac{dV}{dx}\frac{\partial W}{\partial q}-\frac{\hbar^{2}}{3!2^{2}}\frac{d^{3}V}{dx^{3}}\frac{\partial^{3}W}{\partial q^{3}}+\frac{\hbar^{4}}{5!2^{4}}\frac{d^{5}V}{dx^{5}}\frac{\partial^{5}W}{\partial q^{5}}-\cdots , \end{array}$$

with initial condition $W_{\mathrm{in}}$. *W* is real: this can be directly established through either Equation (2) or Equation (6) by taking complex conjugates and changing $x^{\prime }\to x^{\prime \prime }=-x^{\prime }$.

If ℏ→0, then the above equation becomes the classical Liouville equation [1,4,6].

(7) $$\begin{array}{ccc} & & \frac{\partial W(x,q;t)}{\partial t}=-\frac{q}{m}\frac{\partial W(x,q;t)}{\partial x}+\frac{dV}{dx}\frac{\partial W}{\partial q} \end{array}$$

### 2.2. Equilibrium Wigner Function near the Classical Limit

Recall that in the classical case, the equilibrium (or Boltzmann’s) canonical distribution describing the thermal equilibrium of a classical particle with an HB is proportional to a Gaussian in *q*: that is, $\exp\left[-\beta q^{2}/\left(2m\right)\right]$, with *q* now being the classical momentum. In the high temperature or small β (quasiclassical) regime, Wigner [6] obtained successive approximations for $W_{\mathrm{eq}}\left(x,q\right)$ as a power series in *ℏ*. Equations (22) and (25) in [6] (the leading terms in that expansion for $W_{\mathrm{eq}}\left(x,q\right)$) are directly recast as:

(8) $$\begin{array}{ccc} & & W_{\mathrm{eq}}\left(x,q\right)=c_{0}f_{\mathrm{eq}}\left[1+c_{1}+c_{2}\frac{\partial^{2}V}{\partial x^{2}}H_{2}\left(q/q_{eq}\right)\right] \end{array}$$

with

(9) $$\begin{array}{ccc} & & f_{\mathrm{eq}}=\exp\left[-\left(q^{2}/q_{eq}^{2}+\beta V\right)\right] \end{array}$$

(10) $$\begin{array}{ccc} & & c_{1}=\hbar^{2}\left[-\frac{\beta^{2}}{12m}\frac{\partial^{2}V}{\partial x^{2}}+\frac{\beta^{3}}{24m}{\left(\frac{\partial V}{\partial x}\right)}^{2}\right] \end{array}$$

(11) $$\begin{array}{ccc} & & c_{2}=\frac{{\left(\beta \hbar \right)}^{2}}{48m} \end{array}$$

where $c_{0}=2\pi \hbar$. $H_{2}$ denotes the standard Hermite polynomial of second order [38]. We shall refer to the contributions associated with $c_{1}$ and $c_{2}$ as quantum contributions in the quasiclassical regime. Actually, Wigner’s series in [6], after factoring out $c_{0}f_{\mathrm{eq}}$, turns out to also be a power series in β and *q* so that the contributions in his Equation (25) contain only up to quadratic powers in *q*. The latter have been rewritten in terms of $H_{2}$ in (8) in order to facilitate an eventual comparison with $W_{\mathrm{eq}}$ if desired.

In the classical case (ℏ=0) with attractive *V* in some domain, $f_{\mathrm{eq}}$ contains the factor $\exp\left[-\beta V\right]$, which is >1 and can be certainly large in the regions where V<0. Recall that in the classical case, in the latter domains there are no bound states but a continuum of classical orbits. At this point, one may wonder how such a continuum of classical orbits can become a discrete set of bound states if one resorts back to the quantum (statistical) regime of interest here. The above quasiclassical expressions do provide some hint for that. In fact, for $W_{\mathrm{eq}}$ as given in by the small β (or, formally, small *ℏ*) Equation (8), by integrating over *y* (with $y=q/q_{eq}$) and doing some algebra, one has that $\int dyW_{eq}$ tends to:

(12) $$\begin{array}{ccc} & & c_{0}\pi^{1/2}\exp\left[-\beta V\right].\left[1-\hbar^{2}\frac{\beta^{2}}{12m}\frac{\partial^{2}V}{\partial x^{2}}+\frac{\hbar^{2}\beta^{3}}{24m}{\left(\frac{\partial V}{\partial x}\right)}^{2}\right] \end{array}$$

We state that the crucial term $-\hbar^{2}\frac{\beta^{2}}{12m}\frac{\partial^{2}V}{\partial x^{2}}$ is a qualitative signal of how a continuum of classical orbits can become a discrete set of bound states even if it does not provide a quantitative mechanism. In fact, the attractive *V*, being finite and having minima (with values <0) in certain domains, have $\frac{\partial^{2}V}{\partial x^{2}}>0$ at those minima. Then, the leading correction $-\hbar^{2}\frac{\beta^{2}}{12m}\frac{\partial^{2}V}{\partial x^{2}}$ tends to reduce the importance of V(<0) in those domains and contributes to restrict a continuum of classical bounded orbits to a discrete set of quantum bound states. The remaining term $\frac{\hbar^{2}\beta^{3}}{24m}{\left(\frac{\partial V}{\partial x}\right)}^{2}$ (>0) would tend to reinforce $\exp\left[-\beta V\right]$ (with −V(>0)) but at a smaller amount due to the additional factor β and due to the fact that it is small and vanishes at the minimum ($\frac{\partial V}{\partial x}=0$). We shall regard this qualitative behavior as an indication that even the lowest quantum corrections to the classical $f_{\mathrm{re},\mathrm{eq}}$ contain certain, even if small and partial, signals of quantum effects.

### 2.3. $W_{\mathrm{eq}}$ as a Quasi-Definite Functional of Momenta

Notice that neither $W_{\mathrm{eq}}$ nor *W* can be warranted to be nonnegative in general [8,37,39,40]. However, by invoking a suitable extension [41] of the theory of orthogonal polynomials, it is natural: (i) to accept that $W_{\mathrm{eq}}$ is a quasi-definite functional of momenta (as one can justify through examples in certain cases: see references in [29]) and (ii) to invoke that $W_{\mathrm{eq}}$ can be regarded as a generating function to construct recurrently an infinite family of orthogonal polynomials in *q*.

Let $y=q/q_{eq}$. We introduce (unnormalized) orthogonal polynomials $H_{Q,n}=H_{Q,n}\left(y\right)$ in *y* determined by $W_{eq}$, which acts as a (in general, non-Gaussian) weight function. The term *n* is a non-negative integer. $H_{Q,n}\left(y\right)$ also depends parametrically on *x*, although such a dependence is not displayed explicitly. Under the assumptions about *V* in Section 2.1, $W_{\mathrm{eq}}$ is an even function of *q*: this is immediately confirmed, in particular just by looking at the classical and quasiclassical expressions for it given in Section 3.2. Then, $H_{Q,n}$ is even or odd in *y* for even or odd *n*, respectively. We choose $H_{Q,0}=H_{Q,0}\left(y\right)=1$. We also choose: $H_{Q,1}=H_{Q,1}\left(y\right)=y$, $H_{Q,2}=H_{Q,2}\left(y\right)=y^{2}+\epsilon_{2,0}$, $H_{Q,3}=H_{Q,3}\left(y\right)=y^{3}+\epsilon_{3,1}y$, and so on. In general, the $H_{Q,n}$ terms are constructed recurrently as follows. We impose for $n\neq n^{\prime }$ and any *x* (left unintegrated) that

(13) $$\begin{array}{c} \int dyW_{\mathrm{eq}}\left(x,y\right)H_{Q,n}\left(y\right)H_{Q,n^{\prime }}\left(y\right)=0, \end{array}$$

where

(14) $$\begin{array}{ccc} & & H_{Q,n}\left(y\right)=y^{n}+\sum_{j}\epsilon_{n,n-j}y^{n-j}+\cdots \end{array}$$

with 0≤j≤n and n−j=2,4,…. Here, $\epsilon_{n,n-j}$ are dimensionless and *y*-independent (though *x*-dependent, in general). One has $\epsilon_{n,n-j}=0$ if *j* is odd so that $H_{Q,n}\left(-y\right)=\left(-1\right)H_{Q,n}\left(y\right)$.

The nonvanishing $\epsilon_{n,n-2}$ for low-order n=2,3,4,5 (*j* even) are

(15) $$\begin{array}{ccc} & & \epsilon_{2,0}=-\langle y^{2}\rangle ,\;\epsilon_{3,1}=-\frac{\langle y^{4}\rangle }{\langle y^{2}\rangle }, \end{array}$$

(16) $$\begin{array}{ccc} & & \epsilon_{4,2}=\frac{\langle y^{2}\rangle \langle y^{4}\rangle -\langle y^{6}\rangle }{\langle y^{4}\rangle -{\langle y^{2}\rangle }^{2}},\;\epsilon_{4,0}=\frac{\langle y^{2}\rangle \langle y^{6}\rangle -{\langle y^{4}\rangle }^{2}}{\langle y^{4}\rangle -{\langle y^{2}\rangle }^{2}} \end{array}$$

(17) $$\begin{array}{ccc} & & \epsilon_{5,3}=\frac{\langle y^{4}\rangle \langle y^{6}\rangle -\langle y^{2}\rangle \langle y^{8}\rangle }{\langle y^{2}\rangle \langle y^{6}\rangle -{\langle y^{4}\rangle }^{2}}, \end{array}$$

(18) $$\begin{array}{ccc} & & \langle y^{n}\rangle =\frac{\int dyW_{\mathrm{eq}}\left(x,q\right)y^{n}}{\int dyW_{\mathrm{eq}}\left(x,q\right)}. \end{array}$$

They fulfill the following exact quantum identities:

(19) $$\begin{array}{ccc} & & \left(\epsilon_{3,1}-\epsilon_{4,2}\right)\epsilon_{2,0}+\epsilon_{4,0}=0 \end{array}$$

(20) $$\begin{array}{ccc} & & -\left(\epsilon_{4,2}-\epsilon_{5,3}\right)\epsilon_{3,1}+\epsilon_{4,0}-\epsilon_{5,1}=0 \end{array}$$

(21) $$\begin{array}{ccc} & & \epsilon_{5,1}=\frac{\langle y^{4}\rangle \langle y^{8}\rangle -{\langle y^{6}\rangle }^{2}}{\langle y^{2}\rangle \langle y^{6}\rangle -{\langle y^{4}\rangle }^{2}} \end{array}$$

There is an infinite number of identities among higher ϵ values, which lie outside our scope here. In the strict quantum regime, $H_{Q,n}\left(y\right)$ are different from the standard Hermite polynomials. The procedure for successively constructing $H_{Q,n}\left(y\right)$ based upon (13) and (14) becomes increasingly cumbersome as *n* increases, even if it is conceptually straightforward: notice that possible recurrence relations among $H_{Q,n}\left(y\right)$ are still lacking so far. In the classical limit (ℏ→0), with Weq(x,q) approximated by the classical Boltzmann distribution $c_{0}f_{eq}$, the orthogonal polynomials $H_{Q,n}\left(y\right)$ are equal to $2^{-n}H_{n}\left(y\right)$, with $H_{n}\left(y\right)$ being the standard Hermite polynomials [38]. Then, for the latter, the computation of all coefficients in the (classical) counterpart of (14) boils down to that of $\langle y^{n}\rangle$ and the latter to that of Gaussian integrals. In the classical limit, one finds:

(22) $$\begin{array}{ccc} & & \epsilon_{2,0}=-\frac{1}{2},\epsilon_{3,1}=-\frac{3}{2},\epsilon_{4,0}=\frac{3}{4},\epsilon_{4,2}=-3,\epsilon_{5,3}=-5,\epsilon_{5,1}=\frac{15}{4} \end{array}$$

which are *x*-independent. The term $\epsilon_{2,0}$ in the quantum regime will play an important role: it will be studied in Section 2.7.

### 2.4. Non-Equilibrium Moments and Hierarchy

We shall proceed to the non-equilibrium Equations (5) and (6). We shall use the (unnormalized) polynomials in *y* ($=q/q_{\mathrm{eq}}$) $H_{Q,n}=H_{Q,n}\left(y\right)$ (n=0,1,2,3,…) orthogonalized in *y* (for fixed *x*) by using the equilibrium distribution $W_{\mathrm{eq}}$ as a weight function. The actual $H_{Q,n}\left(y\right)$ lead to defining the non-equilibrium moments (n=0,1,2,…):

(23) $$\begin{array}{ccc} & & W_{n}=W_{n}\left(x;t\right)=\int dyH_{Q,n}\left(y\right)W \end{array}$$

The initial moments $W_{\mathrm{in},n}$ for $W_{n}$ are obtained by replacing *W* with $W_{\mathrm{in}}$ in Equation (23). The transformation of the one-dimensional Equations (5) and (6) into a linear hierarchy for the non-equilibrium moments $W_{n}$ will play an important role in this work. It can be carried out through direct computations and cancellations employing Equations (13) and (23), which are increasingly cumbersome as *n* increases. The general (*t*-reversible) hierarchy implied by Equations (5) and (6) for any *n* reads:

(24) $$\begin{array}{ccc} \frac{\partial W_{n}}{\partial t}\;\; & = & \;\;-M_{n,n+1}W_{n+1}-\sum_{n^{\prime }=1}^{n}M_{n,n-n^{\prime }}W_{n-n^{\prime }} \end{array}$$

The *M*’s are *t*-independent operator coefficients, which are characterized below. In general, the quantum hierarchy is not a three-term one. The following operator coefficients for any *n* will play a key role:

(25) $$\begin{array}{ccc} M_{n,n+1}W_{n+1}\;\; & \equiv & \;\;\frac{q_{\mathrm{eq}}}{m}\frac{\partial W_{n+1}}{\partial x} \end{array}$$

(26) $$\begin{array}{ccc} M_{n,n-1}W_{n-1}\;\; & = & \;\;-\frac{q_{\mathrm{eq}}}{m}\left[\left(\epsilon_{n+1,n-1}-\epsilon_{n,n-2}\right)\frac{\partial W_{n-1}}{\partial x}\right]+\frac{n}{q_{\mathrm{eq}}}\frac{\partial V}{\partial x}W_{n-1}-\frac{q_{\mathrm{eq}}}{m}\frac{\partial \epsilon_{n,n-2}}{\partial x}W_{n-1}. \end{array}$$

Thus, in the exact non-equilibrium quantum hierarchy (24), the contributions from $W_{n+1}$ always have the same structure ($-\left(q_{\mathrm{eq}}/m\right)\partial W_{n+1}/\partial x$) for any *n*. Then $M_{n,n+1}$ is *n*-independent.

It is very convenient to perform a Laplace transform of the general hierarchy (24) so as to replace each $W_{n}$ with its Laplace transform ${\tilde{W}}_{n}={\tilde{W}}_{n}\left(s\right)=\int_{0}^{+\infty }dtW_{n}\left(t\right)\exp\left(-st\right)$.

Then, the above hierarchy (24) becomes the Laplace-transformed quantum hierarchy:

(27) $$\begin{array}{ccc} s{\tilde{W}}_{n}\;\; & = & W_{\mathrm{in},n}\;\;-M_{n,n+1}{\tilde{W}}_{n+1}-\sum_{n^{\prime }=1}^{n}M_{n,n-n^{\prime }}{\tilde{W}}_{n-n^{\prime }} \end{array}$$

with the same *t*-independent operators (the *M*’s) as in (24). Notice that $M_{n,n^{\prime }=0}=0$ for any *n* except for n=1, and $M_{n,n-n^{\prime }}=0$ if $n-n^{\prime }$ is even. For simplicity and without any essential loss of generality, we shall assume $W_{\mathrm{in},n}=0$ for ,n>0 while only $W_{\mathrm{in},n=0}\neq 0$.

In detail, the lowest equations in the Laplace-transformed quantum hierarchy (27) are:

(28) $$\begin{array}{ccc} & & s{\tilde{W}}_{0}=W_{\mathrm{in},0}-M_{0,1}{\tilde{W}}_{1} \end{array}$$

(29) $$\begin{array}{ccc} & & s{\tilde{W}}_{1}=-M_{1,2}{\tilde{W}}_{2}-M_{1,0}{\tilde{W}}_{0} \end{array}$$

(30) $$\begin{array}{ccc} & & s{\tilde{W}}_{2}=-M_{2,3}{\tilde{W}}_{3}-M_{2,1}{\tilde{W}}_{1} \end{array}$$

(31) $$\begin{array}{ccc} & & s{\tilde{W}}_{3}=-M_{3,4}{\tilde{W}}_{4}-M_{3,2}{\tilde{W}}_{2} \end{array}$$

(32) $$\begin{array}{ccc} & & s{\tilde{W}}_{4}=-M_{4,5}{\tilde{W}}_{5}-M_{4,3}{\tilde{W}}_{3}-M_{4,1}{\tilde{W}}_{1} \end{array}$$

(33) $$\begin{array}{ccc} & & s{\tilde{W}}_{5}=-M_{5,6}{\tilde{W}}_{6}-M_{5,4}{\tilde{W}}_{4}-M_{5,2}{\tilde{W}}_{2} \end{array}$$

(34) $$\begin{array}{ccc} & & s{\tilde{W}}_{6}=-M_{6,7}{\tilde{W}}_{7}-M_{6,5}{\tilde{W}}_{5}-M_{6,3}{\tilde{W}}_{3}-M_{6,1}{\tilde{W}}_{1} \end{array}$$

and so on for higher values of the integer *n*. A key feature of the non-equilibrium hierarchies (24) and (27) is that all operator coefficients (*M*) in the former are expressed in terms of *V* and of quantities computed out of the equilibrium Wigner function $W_{\mathrm{eq}}$. The $M_{n,n^{\prime }}$ for n=0,1,2,3,4 are identified upon comparing Equation (27) and Equations (28)–(32). Specifically, the operator coefficients (*M*) in the above equations follow directly from:

(35) $$\begin{array}{ccc} s{\tilde{W}}_{0}=W_{\mathrm{in},0} & - & \;\;\frac{q_{\mathrm{eq}}}{m}\frac{\partial {\tilde{W}}_{1}}{\partial x}, \end{array}$$

(36) $$\begin{array}{ccc} s{\tilde{W}}_{1} & =- & \;\;\frac{q_{\mathrm{eq}}}{m}\frac{\partial {\tilde{W}}_{2}}{\partial x}+\frac{q_{eq}}{m}\frac{\partial }{\partial x}\left(\left(\epsilon_{2,0}\right){\tilde{W}}_{0}\right)+\frac{1}{q_{\mathrm{eq}}}\frac{dV}{dx}{\tilde{W}}_{0}, \end{array}$$

(37) $$\begin{array}{ccc} s{\tilde{W}}_{2}\;\; & = & \;\;-\frac{q_{\mathrm{eq}}}{m}\frac{\partial {\tilde{W}}_{3}}{\partial x}+\frac{q_{\mathrm{eq}}}{m}\frac{\partial }{\partial x}\left(\left(\epsilon_{3,1}-\epsilon_{2,0}\right){\tilde{W}}_{1}\right)+\frac{q_{\mathrm{eq}}}{m}\frac{d\epsilon_{2,0}}{dx}{\tilde{W}}_{1}+\frac{2}{q_{\mathrm{eq}}}\frac{dV}{dx}{\tilde{W}}_{1}, \end{array}$$

(38) $$\begin{array}{ccc} s{\tilde{W}}_{3}\;\; & = & \;\;-\frac{q_{\mathrm{eq}}}{m}\frac{\partial {\tilde{W}}_{4}}{\partial x}+\frac{q_{\mathrm{eq}}}{m}\frac{\partial }{\partial x}\left(\left(\epsilon_{4,2}-\epsilon_{3,1}\right){\tilde{W}}_{2}\right)+\frac{q_{\mathrm{eq}}}{m}\frac{d\epsilon_{3,1}}{dx}{\tilde{W}}_{2}+\frac{3}{q_{\mathrm{eq}}}\frac{dV}{dx}{\tilde{W}}_{2}, \end{array}$$

(39) $$\begin{array}{ccc} s{\tilde{W}}_{4}\;\; & = & \;\;-\frac{q_{\mathrm{eq}}}{m}\frac{\partial {\tilde{W}}_{5}}{\partial x}+\frac{q_{\mathrm{eq}}}{m}\frac{\partial }{\partial x}\left(\left(\epsilon_{5,3}-\epsilon_{4,2}\right){\tilde{W}}_{3}\right)+\frac{q_{\mathrm{eq}}}{m}\frac{d\epsilon_{4,2}}{dx}{\tilde{W}}_{3}+\frac{4}{q_{\mathrm{eq}}}\frac{dV}{dx}{\tilde{W}}_{3}\\ \;\; & + & \;\;\frac{\hbar^{2}}{2^{2}q_{\mathrm{eq}}^{3}}\frac{d^{3}V}{dx^{3}}\left(-6+\frac{\epsilon_{4,2}}{\epsilon_{2,0}}\right){\tilde{W}}_{1} \end{array}$$

The $\epsilon_{n,n-2}$ in the first four Equations (35)–(38) do contain inside them (even if not in an explicit way) quantum effects arising from $W_{eq}$.

The derivations of the successive equations in the non-equilibrium recurrence starting from the equation for $s{\tilde{W}}_{3}$ onward are increasingly difficult, as they involve exact cancellations in order to arrive at consistent equations.

Thus, Equation (38) for $s{\tilde{W}}_{3}$ makes use of the identity in (19) and it involves the exact cancellation of the contribution of ${\tilde{W}}_{0}$.

Also, Equation (39) for $s{\tilde{W}}_{4}$ employs the identity in (20) and involves other exact cancellations in such a way that the contribution of ${\tilde{W}}_{1}$ is explicitly proportional to the quantum correction $\frac{\hbar^{2}}{2^{2}q_{\mathrm{eq}}^{3}}\frac{d^{3}V}{dx^{3}}$ (of order $\hbar^{2}$) as it stands. We emphasize that such a contribution multiplying ${\tilde{W}}_{1}$ in Equation (39) for $s{\tilde{W}}_{4}$ has an explicit quantum origin. In that aspect, (39) does differ from (35)–(38). The reason for that difference is that the quantum corrections in Equations (5) and (6) manifest themselves explicitly only at order $\hbar^{2}$ and then, in turn, in the equations in the hierarchy at orders n≥4.

The very fact that the full quantum Equation (39) for n=4 does contain a term of order $\hbar^{2}$ in ${\tilde{W}}_{1}$ confirms that the quantum hierarchy (24) is not a three-term hierarchy.

${\tilde{W}}_{n^{\prime }}$ ($0<n^{\prime }\leq n-1$) do carry *n*-dependent coefficients, which increase with *n*. In particular (and leaving aside other contributions), the equation for $s{\tilde{W}}_{5}$ can be shown to contain in its right-hand-side $\left(\hbar^{2}/q_{\mathrm{eq}}^{3}\right)\left(\partial^{3}V/\partial x^{3}\right){\tilde{W}}_{2}$ as the highest spatial derivative of *V*, while that for $s{\tilde{W}}_{6}$ contains $\left(\hbar^{2}/q_{\mathrm{eq}}^{3}\right)\left(\partial^{3}V/\partial x^{3}\right){\tilde{W}}_{3}$ and $\left(\hbar^{4}/q_{\mathrm{eq}}^{5}\right)\left(\partial^{5}V/\partial x^{5}\right){\tilde{W}}_{1}$ and so on.

We stress that the complicated structure of the general hierarchy (24) is a genuine consequence of quantum mechanics. If ℏ→0, the hierarchy for the classical limit of ${\tilde{W}}_{n}$ becomes the following three-term one for Equation (7):

(40) $$\begin{array}{ccc} \frac{\partial W_{n}}{\partial t}\;\; & = & \;\;-M_{n,n+1}W_{n+1}-M_{n,n-1}W_{n-1} \end{array}$$

(41) $$\begin{array}{ccc} M_{n,n+1}\;\; & \equiv & \;\;\frac{q_{\mathrm{eq}}}{m}\frac{\partial }{\partial x} \end{array}$$

(42) $$\begin{array}{ccc} M_{n,n-1}\;\; & = & \;\;-\frac{q_{\mathrm{eq}}}{m}\left[\left(\epsilon_{n+1,n-1}-\epsilon_{n,n-2}\right)\frac{\partial }{\partial x}\right]+\frac{n}{q_{\mathrm{eq}}}\frac{\partial V}{\partial x}. \end{array}$$

with $M_{n,n-n^{\prime }}=0$ for $n^{\prime }\neq 1$ and the same $M_{n,n+1}$. Specifically, one has:

(43) $$\begin{array}{ccc} & & M_{n,n-1}=\frac{q_{\mathrm{eq}}n}{m}\left[\frac{1}{2}\frac{\partial }{\partial x}+\frac{m}{q_{\mathrm{eq}}^{2}}\frac{\partial V}{\partial x}\right] \end{array}$$

Notice the crucial simplification for ℏ→0: ϵ become independent on *x*.

### 2.5. Formal Solution of General Hierarchy

It is methodologically important and useful to obtain a formal solution of the general quantum hierarchy (24) that is not a three-term one (that is, without proceeding to the classical regime). For that purpose, one solves the hierarchy (24) for n>0 recurrently. Then, all $W_{n}$ for n>0 can be expressed through suitable linear operators in terms of $W_{0}$ and of the initial condition $W_{\mathrm{in},0}$ (assuming $W_{\mathrm{in},n}=0$ for n>0). In particular, it will be important to get $W_{1}$ as a linear functional of $W_{0}$.

In practice, it is convenient to operate in terms of Laplace transforms by employing (27). The solution of the hierarchy proceeds specifically as follows: (i) for suitable $n_{0}\geq 2$, in the equation of the hierarchy (27) yielding ${\tilde{W}}_{n_{0}}\left(s\right)$, one omits ${\tilde{W}}_{n_{0}+1}\left(s\right)$ and solves formally the resulting equation for ${\tilde{W}}_{n_{0}}\left(s\right)$ in terms of those ${\tilde{W}}_{n_{0}-n}\left(s\right)$ for n>0 appearing in the right-hand-side of that equation; (ii) one proceeds to the equation of the hierarchy yielding ${\tilde{W}}_{n_{0}-1}\left(s\right)$, reshuffles into it the above expression for ${\tilde{W}}_{n_{0}}\left(s\right)$, and solves for ${\tilde{W}}_{n_{0}-1}\left(s\right)$; (iii) one proceeds by iteration to the equations for ${\tilde{W}}_{n_{0}-n}\left(s\right)$ for n>1 and so on until one arrives at ${\tilde{W}}_{1}\left(s\right)$ in terms of ${\tilde{W}}_{0}\left(s\right)$. Then, one repeats the above procedure for ${\tilde{W}}_{n_{0}+1}\left(s\right)$ and infers by induction what the formal structure of the solution is as $n_{0}\to +\infty$. One finds (*s*-dependences being understood for brevity as suitable):

(44) $$\begin{array}{ccc} & & {\tilde{W}}_{1}\left(s\right)=D_{1}\left(s\right)\left[-M_{1,0}\right]{\tilde{W}}_{0}\left(s\right). \end{array}$$

(45) $$\begin{array}{ccc} & & D_{1}=\frac{1}{sI-M_{1,2}D_{2}\left(M_{2,1}+M_{2,3}D_{3}M_{3,4}D_{4}M_{4,1}+M_{2,3}D_{3}M_{3,4}D_{4}M_{4,5}D_{5}M_{5,6}D_{6}M_{6,1}+\ldots \right)}, \end{array}$$

(46) $$\begin{array}{ccc} & & D_{2}=\frac{1}{sI-M_{2,3}D_{3}\left(M_{3,2}+M_{3,4}D_{4}M_{4,5}D_{5}M_{5,2}+M_{3,4}D_{4}M_{4,5}D_{5}M_{5,6}D_{6}M_{6,7}D_{7}M_{7,2}+\ldots \right)}, \end{array}$$

(47) $$\begin{array}{ccc} & & D_{3}=\frac{1}{sI-M_{3,4}D_{4}\left(M_{4,3}+M_{4,5}D_{5}M_{5,6}D_{6}M_{6,3}+M_{4,5}D_{5}M_{5,6}D_{6}M_{6,7}D_{7}M_{7,8}D_{8}M_{8,3}+\ldots \right)} \end{array}$$

and so on. The *D* vales are continued-fraction linear operators. *I* is the unit operator. In a compact form:

(48) $$\begin{array}{ccc} & & D_{n}=D_{n}\left(s\right)=\frac{1}{sI-M_{n,n+1}D_{n+1}\left(M_{n+1,n}+N_{n+1,n}\right)} \end{array}$$

(49) $$\begin{array}{ccc} & & N_{n+1,n}=N_{n+1,n}\left(s\right)=M_{n+1,n+2}D_{n+2}M_{n+2,n+3}D_{n+3}M_{n+3.n}+\ldots \end{array}$$

with the understanding that if the infinite continued fraction related to $D_{n}$ in (48) is approximated by a finite continued fraction by cutting it off at some $n_{0}$, the infinite series for $N_{n+1,n}$ is approximated by a finite sum that is cut off at $n_{0}$; thereby, its last term contains $M_{n_{0}.n}$.

Then, one has the exact equation for ${\tilde{W}}_{0}\left(s\right)$:

(50) $$\begin{array}{ccc} & & s{\tilde{W}}_{0}\left(s\right)=W_{\mathrm{in},n}-M_{0,1}D_{1}\left[-M_{1,0}\right]{\tilde{W}}_{0}\left(s\right) \end{array}$$

For previous approaches to stochastic equations that make use of continued fractions, see [42,43]. As a general reference on ordinary continued fractions, see [44]. Notice the genuine cyclic structure (determined by the structure of the hierarchy) in successive terms in the denominators in $D_{n}$. For instance, in $D_{1}$, the contribution $M_{2,3}D_{3}M_{3,4}D_{4}M_{4,1}$ in the series $N_{2,1}$ indeed achieves a cyclic structure in the indices of *M* due precisely to $M_{4,1}=-\frac{\hbar^{2}}{2^{2}q_{\mathrm{eq}}^{3}}\frac{\partial^{3}V}{\partial x^{3}}\left(-6+\frac{\epsilon_{4,2}}{\epsilon_{2,0}}\right)$, which is a quantum correction depending explicitly on *ℏ* (and also implicitly on it through $\frac{\epsilon_{4,2}}{\epsilon_{2,0}}$). In the strict classical limit, $\epsilon_{2,0}=-\left(1/2\right)$ and $\epsilon_{4,2}=-3$, and that quantum correction disappears. And there is similarly with $M_{6,1}$ in $M_{2,3}D_{3}M_{3,4}D_{4}M_{4,5}D_{5}M_{5,6}D_{6}M_{6,1}$ and so on.

There are other approaches (different from the one pursued here, to the best of the present authors’ knowledge) to the analysis of general linear recurrence relations: see, for instance [45].

### 2.6. Convergence: A Qualitative Analysis

A detailed mathematical study lies outside the scope of this work, as we shall limit ourselves to indicate arguments justifying convergence. We shall consider first the case ℏ→0. Then, one has the three-term hierarchy in Equations (40), (41) and (43) for the Liouville equation. Equation (48) still holds with the series $N_{n+1,n}\to 0$ so that

(51) $$\begin{array}{ccc} & & D_{n}=D_{n}\left(s\right)=\frac{1}{sI-M_{n,n+1}D_{n+1}M_{n+1,n}} \end{array}$$

with $M_{n,n+1}$ and $M_{n+1,n}$ being given in Equations (41) and (43). We shall introduce the *n*-independent operators

(52) $$\begin{array}{ccc} & & F=M_{n,n+1}\left[\left(-M_{n+1,n}\right)/\left(n+1\right)\right] \end{array}$$

(53) $$\begin{array}{ccc} & & a_{+}=\frac{1}{F^{1/4}}M_{n,n+1}\frac{1}{F^{1/4}},a_{-}=\frac{1}{F^{1/4}}\left[\left(-M_{n+1,n}\right)/\left(n+1\right)\right]\frac{1}{F^{1/4}} \end{array}$$

Let s=0 in Equation (48) and iterate it indefinitely. By using Equations (52) and (53), a direct formal computation yields:

(54) $$\begin{array}{ccc} & & D_{n}\left(0\right)=2^{-1/2}\frac{\Gamma ((n/a+-2)+1/2)}{\Gamma ((n/2)+1)}F^{-1/4}AF^{-1/4},A=\frac{1}{a_{-}Aa_{+}} \end{array}$$

where Γ is the standard Gamma function [46] and the operators *F* and *A* being *n*-independent. The following formal and short discussion (simplifying unimportant dependences for it) may be adequate. Let us assume the operator $F=-\frac{\partial }{\partial x}\left(a\frac{\partial }{\partial x}+b\right)$ with real functions a=a(x)≥0 and b=b(x) acting on functions f=f(x). Upon transforming f→g with f=exp(−b/a)g, $F\to F_{1}$ with $F_{1}=-\left[\frac{\partial }{\partial x}-\left(b/2a\right)\right]a\left[\frac{\partial }{\partial x}+\left(b/2a\right)\right]$. The operator $F_{1}$ (acting on *g*) is non-negative. The preceding argument is closely related to the one to be employed later in Section 2.9 regarding (62). Going backwards, that enables us to give a meaning to $F^{-1/4}$, $a_{+}$, and $a_{-}$. *A* can be formally regarded as an operator-continued fraction. Even if a rigorous characterization for it is lacking at present, Equation (54) indicates that $D_{n}\left(0\right)$ factorizes into the *n*-independent operator $F^{-1/4}AF^{-1/4}$ times an explicit function of *n*. For large *n*, $\frac{\Gamma ((n/2)+1/2)}{\Gamma ((n/2)+1)}$ behaves as $2n^{-1/2}$ [44] and $D_{n}\left(0\right)$ tends to vanish proportionally to $n^{-1/2}$. This suggests that the operator $D_{n}\left(s\right)$ in Equation (51) also tends to vanish as $n^{-1/2}$ for large *n*. We shall employ this large *n* behavior in order to assess, for the general hierarchy (24), the convergence of $D_{n}\left(s\right)$ and $N_{n+1,n}\left(s\right)$ in Equations (48) and (49), respectively. That is, in these assessments for continued fractions and infinite series in the exact solutions, we estimate that the exact operator $D_{n}\left(s\right)$ behaves for large *n* and fixed and finite *s* as $n^{-1/2}$ (times an *n*-independent operator). The application of such estimates to the operators $N_{n+1,n}\left(s\right)$ and some judicious guess for the last factor in each operator contribution ($M_{n+3,n}$ in the first contribution in (49) and so on) leads us to infer that the operator $N_{n+1,n}$ is more negligible compared to $M_{n+1,n}$ the larger *n* is. Then, upon estimating $D_{n+1}$ in the right-hand-side of (48) to be of order $n^{-1/2}$ and noticing that $M_{n+1,n}$ is of order *n*, it follows that $D_{n}$ in the left-hand-side of (48) is of order $n^{-1/2}$ consistently. This suggests that $D_{1}$ in Equation (50) is finite. Even if full mathematical justifications are lacking, it is felt that the arguments above provide reasonable support towards the finiteness and well-posedness of the present developments based upon non-equilibrium moments and hierarchy.

### 2.7. Properties at Equilibrium: $\epsilon_{2,0}$

The equilibrium Wigner function in (4) and the definitions of the equilibrium moments using Weq(x,q) as the generating function implies that all $W_{n}=W_{eq,n}=0$ for n=1,2,3,…, while only the lowest one is non-vanishing:

(55) $$\begin{array}{ccc} & & W_{0}=W_{eq,0}=\frac{1}{q_{eq}}\sum_{j}\exp\left[-\beta E_{j}\right]\varphi_{j}\left(x\right)\varphi_{j}^{*}\left(x\right) \end{array}$$

In this case, $W_{\mathrm{in},n}=0$ for any n=0,1,2,…. On the other hand, one has the general equation:

(56) $$\begin{array}{ccc} & & M_{1,0}W_{n}=-\frac{q_{eq}}{m}\frac{\partial }{\partial x}\left(\left(\epsilon_{2,0}\right)W_{n}\right)+\frac{1}{q_{\mathrm{eq}}}\frac{\partial V}{\partial x}W_{n} \end{array}$$

Equation (24) for equilibrium and n=1 imply

(57) $$\begin{array}{ccc} & & M_{1,0}W_{eq,0}=0 \end{array}$$

Equation (57) and the expression for $M_{1,0}$ from (56) with the condition that in the classical (ℏ→0) limit, $-\epsilon_{2,0}=1/2$, can be recast as two alternative and exact representations, each having its own interest. First, Equation (57) implies:

(58) $$\begin{array}{ccc} & & -\epsilon_{2,0}\left(x\right)W_{eq,0}\left(x\right)=\left(1/2\right)\exp\left[-\frac{m}{q_{eq}^{2}}\int_{x}^{+\infty }dx^{\prime }\frac{1}{\epsilon_{2,0}\left(x^{\prime }\right)}\frac{\partial V}{\partial x^{\prime }}\right], \end{array}$$

Since $W_{eq,0}\geq 0$, it follows that $-\epsilon_{2,0}\geq 0$ for any *x*.

Second, an explicit representation of $-\epsilon_{2,0}$ in terms of $W_{eq,0}$ and *V* is:

(59) $$\begin{array}{ccc} & & \epsilon_{2,0}\left(x\right)=-\frac{1}{2W_{eq,0}\left(x\right)}-\frac{m}{q_{eq}^{2}W_{eq,0}\left(x\right)}\int_{x}^{+\infty }dx^{\prime }W_{eq,0}\left(x^{\prime }\right)\frac{\partial V}{\partial x^{\prime }}, \end{array}$$

The formally infinite integration limits in Equations (58) and (59) are to be understood in the framework (very large *L*) and conditions on *V* stated in Section 2.1. In particular, since *V* is appreciable in a finite interval (−a<x<a), the integrals in Equations (58) and (59) converge.

### 2.8. One-Dimensional Non-Equilibrium Hierarchy: Small Thermal Wavelength

Let δx be a typical scale of variation of *V*: for instance, a fraction (say, 1/5 to 1/10) of one nanometer. We shall consider a quantum regime with a relatively small thermal wavelength with, say, $\lambda_{th}/\delta x<1$: some features in it are treated as in the classical regime while others behave quantum mechanically. In such a regime, chemical reactions of the kind we are interested in occur. Such a regime is not the strict classical high-temperature limit. For assumptions and estimates characterizing such a regime, see Section 3.2 in [30]. A summary of those estimates is the following. Let the mass *m* be about one to two orders of magnitude larger than the neutron mass. The range of *V* may be about 0.1 to 0.5 nanometers. The value of $k_{B}T$ may lie, for instance, between 300 K (room temperature) and, say, 1200 K. ∣V∣ may vary between 1 and a few eV, and let $V_{0}$ be a positive constant energy having a value in such a range. Let $\frac{\partial^{n}V}{\partial x^{n}}$ be estimated as $\frac{\delta^{n}V}{{\left(\delta x\right)}^{n}}$. In turn, δV is estimated as one order of magnitude smaller than $V_{0}$, and $\delta^{n}V$ is estimated as one order of magnitude smaller than $\delta^{n-1}V$, n=2,3,….

In Equation (39), $\frac{\hbar^{2}}{q_{\mathrm{eq}}^{3}}\frac{\partial^{3}V}{\partial x^{3}}$ appears to be smaller than $\frac{1}{q_{\mathrm{eq}}}\frac{\partial V}{\partial x}$ by a factor ${\left(\frac{\lambda_{\mathrm{th}}}{\delta x}\right)}^{2}$ times a contribution smaller than unity. This suggests we can neglect the quantum correction containing $\frac{\hbar^{2}}{2^{2}q_{\mathrm{eq}}^{3}}\frac{\partial^{3}V}{\partial x^{3}}\left[-6+\frac{\epsilon_{4,2}}{\epsilon_{2,0}}\right]$ compared to the one containing $\frac{1}{q_{\mathrm{eq}}}\frac{\partial V}{\partial x}$ in Equation (39). Similar approximations can be carried out in the equation for $\partial W_{5}/\partial t$ (by neglecting the contribution containing $\left(\hbar^{2}/q_{\mathrm{eq}}^{3}\right)\partial^{3}V/\partial x^{3})$ and in the equation for $\partial W_{6}/\partial t$ (by neglecting $\left(\hbar^{2}/q_{\mathrm{eq}}^{3}\right)\left(\partial^{3}V/\partial x^{3}\right)$ and $\left(\hbar^{4}/q_{\mathrm{eq}}^{5}\right)\left(\partial^{5}V/\partial x^{5}\right)$) and so on. In general, we shall accept as a plausible approximation (applicable in applications for chemical reactions) that in Equation (24), one can neglect, on average, all contributions due to all $M_{n,n-n^{\prime }}W_{n^{\prime }}$ with $n^{\prime }=2,\ldots n-1$ compared to $M_{n,n-1}W_{n-1}$. Then, Equation (24) becomes the approximate (*t*-reversible) three-term hierarchy

(60) $$\begin{array}{c} \frac{\partial W_{n}}{\partial t}=-M_{n,n+1}W_{n+1}-M_{n,n-1}W_{n-1}, \end{array}$$

but we still retain the full $M_{n},n-1$ (including its own quantum corrections) in Equation (26). The latter makes Equation (60) differ from Equation (40), in which $M_{n},n-1$ is given in Equation (43).

Alternatively, in $D_{1}$, the contribution $M_{2,3}D_{3}M_{3,4}D_{4}M_{4,1}$ (with a cyclic structure in the indices of *M*) is smaller than $M_{2,1}$ thanks formally to the $\hbar^{2}$ in the contribution $M_{4,1}$. In physical terms, we proceed to the quantum regime with a relatively small thermal wavelength as characterized above: then, on average, the magnitude of the values implied by the action of the operator $M_{2,3}D_{3}M_{3,4}D_{4}M_{4,1}$ on a generic function may be smaller (by about one to two orders of magnitude) than those by the operator $M_{2,1}$. Then, in $D_{1}$, it appears plausible to discard the contribution $M_{2,3}D_{3}M_{3,4}D_{4}M_{4,1}$ and, by a similar argument, the full $M_{2,3}D_{3}M_{3,4}D_{4}M_{4,5}D_{5}M_{5,6}D_{6}M_{6,1}+\ldots$ compared to $M_{2,1}$. It is stressed that the full $M_{2,1}$ is still kept (thereby still taking into account certain quantum effects). The actual counterpart to Equation (48) for the three-term hierarchy (60) is, for n=1,2,3…:

(61) $$\begin{array}{ccc} & & D_{n}=D_{n}\left(s\right)=\frac{1}{sI-M_{n,n+1}D_{n+1}M_{n+1,n}} \end{array}$$

That is consistent with the operator-continued fractions that follow directly from Equation (60). We shall continue to assume the initial condition $W_{in,0}\neq 0$, $W_{in,0}\neq W_{eq,0}$, and $W_{in,n}=0$ for n≠0 for simplicity. This amounts to discarding all contributions in the operators containing cyclic structures: that is, to discard $M_{2,3}D_{3}M_{3,4}D_{4}M_{4,5}D_{5}M_{5,6}D_{6}M_{6,1}+\ldots$ in $D_{1}$ and so on for $D_{n}$ for n=2,3,….

The following remark can be regarded as a gratifying check of consistency. At very high temperatures, practically in the classical regime, and based upon [6], we shall approximate the one-dimensional equilibrium quantum distribution to a leading order by the classical distribution: $W_{\mathrm{eq}}≃c_{0}f_{\mathrm{eq}}$ with $f_{\mathrm{eq}}=\exp\left[-\beta (\left(q^{2}/2m\right)+V)\right]$, thereby neglecting the corrections computed in [6]. Then, the computations of all $\epsilon_{n,n-j}$ boil down to computing Gaussian integrals. From (15) and (16), one easily finds: $\epsilon_{2,0}=-1/2$ and $\epsilon_{4,2}=-3$. Then, under that approximation corresponding to the classical regime, one finds $-6+\frac{\epsilon_{4,2}}{\epsilon_{2,0}}=0$ in Equation (39) and, consequently, the hierarchy Equations (35)–(39) reduce to a three-term one. The same reduction of Equation (24) to a three-term hierarchy occurs for any *n*.

Notice as well the following behavior. Consider the Laplace transform of Equation (60), which implies ${\tilde{W}}_{n}\left(s\right)=-D_{n}M_{n,n-1}{\tilde{W}}_{n-1}\left(s\right)$ for n≥1. For fixed and finite *s* and large *n*, $D_{n}$ behaves as $n^{-1/2}$. Then, $s{\tilde{W}}_{n}\left(s\right)$ is subdominant compared to $M_{n,n+1}{\tilde{W}}_{n+1}\left(s\right)+M_{n,n-1}{\tilde{W}}_{n-1}\left(s\right)$. In turn, that behavior will be consistent with the long-*t* approximation in the next subsection.

### 2.9. One-Dimensional Non-Equilibrium Hierarchy: Long-Time Approximation

We shall proceed to approximations adequate for a long-time non-equilibrium evolution based upon Equation (60), with chemical reactions in mind.

The operators $M_{n,n+1}$ and $M_{n,n-1}$ in (60) have dimensions ${\left(\mathrm{time}\right)}^{-1}$.

The characteristic or effective evolution times associated with those operators, denoted as ${\left(\tau^{*}\right)}^{-1}$, have orders of magnitude that can be estimated easily. Thus, the $\tau^{*}$ associated with $M_{n,n+1}$ is about $\lambda_{\mathrm{th}}m\left(\delta x\right)/\hbar )$ and so on for the various terms contributing to $M_{n,n-1}$ (the estimates of which depend on *x*).

We shall consider *t* about and larger than those effective evolution times. Then, as large *t* corresponds to small *s*, the simplest (long-time) approximation can be formally conjectured for each n=1,2,… as follows: We replace $D_{n}\left(s\right)$ in Equation (61) with the *s*-independent operator $D_{n}\left(s=\epsilon \right)$ (with fixed and small s=ϵ>0), and then, one has the approximation: ${\tilde{W}}_{n}\left(s\right)≃D_{n}\left(s=\epsilon \right)\left[-M_{n,n-1}\right]{\tilde{W}}_{n-1}$ (also regarded as a short-memory approximation). The system formed by the inverse Laplace transform of ${\tilde{W}}_{1}\left(s\right)≃D_{1}\left(s=\epsilon \right)\left[-M_{1,0}\right]{\tilde{W}}_{0}$ together with Equation (60) for n=0 completes the long-time approximation scheme. This amounts to the argument that the *t*-dependence of $W_{n}\left(t\right)$, n=1,2,… would be approximately enslaved by that of $W_{n-1}\left(t\right)$. That immediately yields the following quantum equation:

(62) $$\begin{array}{c} \frac{\partial W_{0}}{\partial t}=\frac{q_{\mathrm{eq}}}{m}\frac{\partial }{\partial x}\left[D_{1}\left(s=\epsilon \right)M_{1,0}W_{0}\right] \end{array}$$

with the above initial condition $W_{in,0}$. Providing a suitable approximation method or ansatz yielding $D_{1}\left(s=\epsilon \right)$ is a difficult open problem: see [30]. The diffusion-like Equation (62) appears to be *t*-irreversible. However, at the present stage, we do not warrant that all eigenvalues of $-\frac{q_{\mathrm{eq}}}{m}\frac{\partial }{\partial x}D_{1}\left(s=\epsilon \right)\left[-M_{1,0}\right]$ are nonnegative.

For the sake of a complementary understanding, we shall accept as a plausible approximation that the linear operator $D_{1}\left(s=\epsilon \right)$ be replaced by a non-negative function $D=D_{1}\left(s=\epsilon \right)\geq 0$ of *x*.

We introduce $f_{0}\left(x;t\right)$ through:

(63) $$\begin{array}{ccc} & & W_{0}=\exp\left[\int_{0}^{x}dx^{\prime }u\left(x^{\prime }\right)\right]f_{0}, \end{array}$$

(64) $$\begin{array}{ccc} & & u\left(x\right)=-\frac{q_{\mathrm{eq}}/m)\left(\partial \left(-\epsilon_{2,0}\right)/\partial x\right)+\left(1/q_{\mathrm{eq}}\right)\left(\partial V/\partial x\right)}{\left(2q_{\mathrm{eq}}/m\right)\left(-\epsilon_{2,0}\right)}. \end{array}$$

Then, Equation (62) becomes

(65) $$\begin{array}{ccc} & & \frac{\partial f_{0}}{\partial t}=\left[\frac{\partial }{\partial x}+u\left(x\right)\right]\frac{q_{\mathrm{eq}}^{2}D\left(-\epsilon_{2,0}\right)}{m^{2}}\left[\frac{\partial }{\partial x}-u\left(x\right)\right]f_{0}. \end{array}$$

Recall that $-\epsilon_{2,0}$ is nonnegative for any *x* (Section 2.7). Then, all eigenvalues of $\left[\frac{\partial }{\partial x}+u\left(x\right)\right]\frac{q_{\mathrm{eq}}^{2}D\left(-\epsilon_{2,0}\right)}{m^{2}}\left[\frac{\partial }{\partial x}-u\left(x\right)\right]$ are nonnegative, and the solution of Equation (65) tends towards $W_{\mathrm{eq},0}$ for t→+∞ for any $W_{\mathrm{in},0}$ (irreversibility and thermalization).

Regarding stochastic equations, see [18,31,47,48,49].

The 1D developments in this section will be very useful at a certain stage (Section 4.4 and Section 4.5) in the 3D model for polymerization explained in the following sections.

## 3. Towards a Model for Catalyzed Polymerization

Throughout this and the following section, we consider a fluid at rest in thermal equilibrium at absolute temperature *T* in an interval about room temperature and in three-dimensional (3D) space. The fluid plays the role of an HB. Then, we consider three ensembles immersed in the fluid: (1) an ensemble of widely separated independent unbound units (atoms and/or small molecules); (2) an ensemble of widely separated, independent, freely jointed (fj) chains as templates (te); and (3) an ensemble of widely separated independent catalysts.

### 3.1. Ensemble of Widely Separated Independent Unbound 3D Units

Let an ensemble of identical $N_{euu}$ non-relativistic microscopic units (atoms and/or small molecules) with equal masses be widely separated from and interacting negligibly with one another. To fix the idea, we concentrate on one of them, denoted as “1”, with mass $M_{1}$, position vector $R_{1}$, momentum $\Pi_{1}$(=−$i\hbar \nabla_{1}$), and quantum kinetic energy $\Pi_{1}^{2}/2M_{1}$, $\nabla_{1}$ as the 3D gradient operator with respect to $R_{1}$. Any wavefunction of the ensemble of $N_{euu}$ units factorizes into the product of $N_{euu}$ individual wavefunctions.

The 3D scalar product of two individual wavefunctions $\psi_{j}$, j=1,2 for the same unit 1 that depends on the same $R_{1}$ reads:

(66) $$\begin{array}{ccc} & & {\left(\psi_{1},\psi_{2}\right)}_{3}\equiv \int d^{3}R_{1}\psi_{1}^{*}\psi_{2}\;, \end{array}$$

with the integration being carried out over $R_{1}$, and * denoting the complex conjugate.

### 3.2. Ensemble of 3D Freely Jointed (fj) Chains as Templates (te)

We consider, also immersed in the fluid, a very dilute solution of identical te molecular chains described below that are adequately separated (and then independent) from one another. That is, the fluid contains an ensemble of such template chains. Any chain is supposed to be adequately long. Regarding molecular chains from various standpoints, see [21,23,31,32,50,51,52,53,54,55].

This subsection will remind the reader of a model for a single 3D te formed by $N_{te}-1$ non-relativistic atoms or small molecules as an open, linear, freely jointed (fj) molecular chain. Let $R_{te,i}$, ${¶}_{te,i}$, and $M_{te,i}$ be the position and momentum vectors and the mass, respectively, of the *i*-th atom in the te ($i=2,\ldots ,N_{te}$). The total mass of the te is: $M_{te}=\sum_{i=2}^{N_{te}}M_{te,i}$. Let $P_{te,CM}=\sum_{i=2}^{N_{te}}P_{te,i}$ be the total momentum operator of the te, and let $y_{i}=R_{te,i+1}-R_{te,i}$, $i=2,\ldots ,N_{te}-1$ be the relative position vectors (the bond vectors) along the te. The latter is treated in the framework of the Born–Oppenheimer approximation [56] so that the most rapidly varying electronic degrees of freedom have already been integrated out. $E_{el}\left(<0\right)$ is the electronic energy (essentially, a constant), which will always be subtracted out from the outset.

In 3D spherical coordinates, the three-momentum operator associated with $y_{i}$ reads:

(67) $$\begin{array}{ccc} & & -i\hbar \nabla_{y_{i}}=-\frac{a_{3,i}}{y_{i}}-i\hbar u_{i}\frac{\partial }{\partial y_{i}}\;, \end{array}$$

with

(68) $$\begin{array}{ccc} & & y_{i}=y_{i}u_{i}\;,a_{3,i}=i\hbar u_{\theta_{i}}\frac{\partial }{\partial \theta_{i}}+i\hbar u_{\varphi_{i}}\frac{1}{\sin\theta_{i}}\frac{\partial }{\partial \varphi_{i}}\;, \end{array}$$

with the three orthonormal vectors:

(69) $$\begin{array}{ccc} & & u_{i}=\left(\cos\varphi_{i}\sin\theta_{i},\sin\varphi_{i}\sin\theta_{i},\cos\theta_{i}\right)\;, \end{array}$$

(70) $$\begin{array}{ccc} & & u_{\theta_{i}}=\left(\cos\varphi_{i}\cos\theta_{i},\sin\varphi_{i}\cos\theta_{i},-\sin\theta_{i}\right)\;, \end{array}$$

(71) $$\begin{array}{ccc} & & u_{\varphi_{i}}=\left(-\sin\varphi_{i},\cos\varphi_{i},0\right) \end{array}$$

Let:

(72) $$\begin{array}{ccc} & & e_{3,l}\equiv i\hbar u_{l}-a_{3,l}, \end{array}$$

$l=2,\ldots ,N_{te}-1$ [53,54,55]. The total quantum kinetic energy of the te is:

(73) $$\begin{array}{ccc} & & P_{te,CM}^{2}/2M_{te}+\sum_{i,j=2}^{N_{te}-1}A_{ij}\left[-i\hbar \nabla_{y_{i}}\right]\left[-i\hbar \nabla_{y_{j}}\right] \end{array}$$

The constants $A_{ij}$ are given by: $M_{i}^{-1}+M_{i+1}^{-1}$ if i=j, $-M_{i}^{-1}$ if j=i−1, $-M_{j}^{-1}$ if j=i+1, and 0, otherwise. So: $A_{ij}=A_{ji}$.

Approximate models for a te as a 3D molecular chain with constrained distances (bond lengths) between successive pairs of neighbor atoms due to strong harmonic-oscillator-like vibrational potentials (covalent bonds that are not as strong as electrical degree-of-freedom interactions) have been constructed [33,53,54,55]. In short, the te is modeled as a freely jointed, or fj, molecular chain. For the sake of a short justification of the latter, as a dominant effective approximation of the covalent bonding (neglecting other weaker interactions), let nearest-neighbor atoms interact through harmonic-oscillator-like potentials with vibrational frequencies $\omega_{0,j}$ [53]. The vibrational energies $\hbar \omega_{0,j}$ (much smaller than $E_{el}$) are supposed to be larger than $K_{B}T$ [53,54,55]. One is also assuming that, on that energy scale, angular degrees of freedom are not constrained. In such a regime, $y_{j}$ equals, approximately, the constant equilibrium distance $d_{j}$ (bond length).

One can also entertain other molecular te with additional constrained distances between successive pairs of next-to-nearest-neighbor units due to (somewhat weaker) harmonic-oscillator-like vibrational potentials: freely rotating molecular chains. Freely rotating chains can be approximated by fj ones that include persistent lengths (namely, effective bond lengths, which amount to constraining approximately both the above $y_{j}$ and also the angles between neighboring bond vectors) [21]. Such models do provide useful approximations for real single-polymer chains under various conditions [21]. In the present work, dealing with fj chains will suffice, with the understanding that the bond lengths can be interpreted as either fixed bond lengths in fj chains or as persistent lengths in freely rotating chains.

Let θ, φ denote, collectively, the two sets $\theta_{2},\ldots ,\theta_{N_{te}-1}$, $\varphi_{2},\ldots ,\varphi_{N_{te}-1}$, respectively. Different 3D variational computations [33,53,54,55] enable us to consistently derive the same 3D model for an fj molecular chain with fixed $y_{j}=d_{j}$ while allowing for purely angular motion of the bond vectors. From those coinciding results, by omitting the total zero-point energy $E_{zp}$ of the vibrations and factoring out the overall center-of-mass motion, one arrives at the following 3D quantum purely angular Hamiltonian and at the scalar product for the fj molecular chain:

(74) $$\begin{array}{ccc} & & {\tilde{H}}_{3,fj}=\sum_{i,j=2}^{N_{te}-1}\frac{A_{ij}}{2d_{i}d_{j}}e_{3,i}e_{3,j} \end{array}$$

(75) $$\begin{array}{ccc} & & {\left(\psi_{1},\psi_{2}\right)}_{3,fj}\equiv \int {\left[d\Omega \right]}_{3}\psi_{1}{\left(\theta ,\varphi \right)}^{*}\psi_{2}\left(\theta ,\varphi \right)\;, \end{array}$$

with ${\left[d\Omega \right]}_{3}=\Pi_{i=2}^{N_{te}-1}d\theta_{i}d\varphi_{i}$. The integration is carried out over the whole $N_{te}-2$ set of solid angles. The angular motion of the te is described by wavefunctions ψ=ψ(θ,φ) and eigenfunctions of the stationary Schrodinger equation, with energy eigenvalues *E*:

(76) $$\begin{array}{ccc} & & {\tilde{H}}_{3,fj}\psi =E\psi \end{array}$$

The purely angular motions described by ${\tilde{H}}_{3,fj}$ will be essential for the understanding of what follows.

The analysis in the remainder of this subsection is intended only as a methodological and essential step towards the study of polymerization in Section 4.

So, we suppose that $K_{B}T$ is adequately smaller than all $\hbar \omega_{0,i}$ and that all vibrational states of any fj chain are the ground-state ones, so that the relevant degrees of freedom of the fj chain are the rotational ones (say, in principle, those corresponding to ${\tilde{H}}_{3,fj}$).

The evolution of an individual fj chain in the fluid at $K_{B}T$ can be described in principle by a non-equilibrium Wigner distribution and equation. However, due to the influence of the fluid at such $K_{B}T$, it will be physically adequate to approximate the quantum description of the rotational motions of the template fj chain as provided by classical statistical mechanics and, hence, by classical Liouville distribution functions.

For units $2,\ldots ,N_{te}$ forming the template fj chain, the approximate transition to classical mechanics reads: $e_{3,i}\to -a_{3,c,i}$, $-i\hbar \frac{\partial }{\partial \theta_{i}}\to \pi_{\theta_{i},c}$, $-i\hbar \frac{\partial }{\partial \varphi_{i}}\to \pi_{\varphi_{i},c}$. So, $-a_{3,c,i}=\pi_{\theta_{i},c}u_{\theta_{i}}+\pi_{\varphi_{i},c}u_{\varphi_{i}}$. The small terms proportional to iℏu are taken as negligible and, so, are disregarded. The terms $\pi_{\theta_{i},c}$, $\pi_{\varphi_{i},c}$ are classical momenta and are canonically conjugate to $\theta_{i}$, $\varphi_{i}$, respectively. The terms $\pi_{\theta ,c}$, $\pi_{\varphi ,c}$ denote the set of all $\pi_{\theta_{i},c}$, $\pi_{\varphi_{i},c}$, respectively.

Then, the quantum Hamiltonian ${\tilde{H}}_{3,fj}$ for the angular motion of the te chain is approximated by the following classical one (factoring out the center-of-mass motion):

${\tilde{H}}_{3,fj,c}=\sum_{i,j=2}^{N_{te}-1}\left(A_{ij}/2d_{i}d_{j}\right)\left(-a_{3,c,i}\right)\left(-a_{3,c,j}\right)$. Let $f_{te,c}=f_{te,c}\left(\theta ,\varphi ,\pi_{\theta ,c},\pi_{\varphi ,c};t\right)$ be the non-equilibrium classical Liouville distribution function for the te. Accordingly, ${\left[{\tilde{H}}_{3,fj,c},f_{te,c}\right]}_{Pb}$ denotes the standard classical Poisson bracket (Pb) of ${\tilde{H}}_{3,fj,c}$ and $f_{te,c}$ [57]. The non-equilibrium classical Liouville equation is:

(77) $$\begin{array}{ccc} & & \frac{\partial f_{te,c}}{\partial t}={\left[{\tilde{H}}_{3,fj,c},f_{te,c}\right]}_{Pb} \end{array}$$

Let ${\left[d\Omega \right]}_{3,L}=\Pi_{i=2}^{N_{te}-1}d\theta_{i}d\pi_{\theta_{i},c}d\varphi_{i}d\pi_{\varphi_{i},c}$. Total probability is consistently conserved: $\partial \int {\left[d\Omega \right]}_{3,L}f_{te,c}/\partial t=0$. These classical approximations for the te chain are instrumental and will be implemented directly in Section 4.1 and Section 4.2.

### 3.3. Ensemble of Widely Separated Independent 3D Catalyst (cat) Molecules

Let an ensemble of identical non-relativistic individual catalyst units be widely separated from (with negligible interactions with) one another. Each catalyst unit (*cat* for short) is a medium-size molecule (formed by other small molecules, monomers…). The total mass of a *cat* unit is $M_{0}$.

The fluid is at rest and in thermal equilibrium at a $K_{B}T$ adequately smaller than all vibrational energies of a *cat* molecule. Then, by assumption, all relevant vibrational states in the *cat* are the ground ones, and the relevant degrees of freedom in it are the rotational ones and those associated with its corresponding center of mass.

With an enormous simplification, at the given *T*, the *cat* is modeled as a slow and free massive molecule of mass $M_{0}$ with position vector $R_{0}$, which approximates the location of its center-of-mass and disregards the spatial extension of the catalyst. Alternatively, $R_{0}$ can also be considered approximately by assumption as the location of a quite reduced domain of the catalyst (the “active” site or center), which will interact with the individual unit and the relevant part of the template and without taking into account the spatial extension of the *cat*.

To fix the ideas, in a quantum-mechanical setting, let $\Pi_{3,0}$ be the quantum momentum canonically conjugate to $R_{0}$. The quantum Hamiltonian of the free catalyst is ${\tilde{H}}_{3,enz}≃\Pi_{3,0}^{2}/2M_{0}$. We advance that from Section 4.1 onward, at the given *T*, it will suffice to approximate the behavior of the *cat* by employing classical mechanics.

## 4. 3D Catalyzed Polymerization of One Single Atom by a Chain: Mixed Quantum–Classical Description

We shall consider a model for chain growth through polymerization: technically, for what is known as insertion polymerization. A (slightly simplified) example is: $\cdots -{\left(CHR-CH_{2}\right)}_{n}-\left[Ti\right]+\left(CHR-CH_{2}\right)\to \cdots -{\left(CHR-CH_{2}\right)}_{n}-\left(CH_{2}-CHR\right)-\left[Ti\right]$. $\left(CHR-CH_{2}\right)$ plays the roles of the individual "small" molecule 1 and of one “small” molecule in the te chain (denoted, in turn, as $\cdots -{\left(CHR-CH_{2}\right)}_{n}$). [Ti], symbolizing, in short, the complex $TiCl_{4}-AIR_{3}$), is the catalyst. See [32].

We suppose that the number of unbound units $N_{euu}$ is approximately equal both to the number of template chains and also to the number of individual *cat* molecules. Then, the fluid at equilibrium and at rest can also be regarded approximately as an ensemble formed by copies adequately separated from one another: with each copy, in turn, being a triplet formed by one unbound unit, one fj chain, and one *cat* molecule. We consider that each copy has a finite volume, albeit it is quite large on the microscopic scale. Those three entities are now regarded as not separated from one another, on average, so that interactions among them occur. Those interactions will be modeled in what follows.

The action of the catalyst enables an unbound unit to become attached to the fj te chain so as to give rise to another larger chain made up of $N_{te}$ atoms. The individual unit is treated quantum-mechanically since its binding to the te is a chemical reaction.

### 4.1. Interactions of a Unit, an fj Chain, and a Catalyst

An individual unit (certainly influenced by its interaction with the catalyst and with the te fj chain) is dealt with quantum-mechanically, as this genuinely applies to its binding process to the template fj chain. In principle, the dynamics are accounted for by a quantum Wigner function [4,6,37].

We introduce the relative vector from that unit to the first atom in the template: $y_{1}=R_{te,2}-R_{1}$. We suppose a rotational-invariant interaction potential between the individual unit and the first atom in the te chain: $U_{1}\left(y_{1}\right)=U_{1}\left(y_{1}\right)$ ($y_{1}=|y_{1}|$). This is appreciable in $0\leq y_{1}\leq y_{1,2}$. Specifically, $U_{1}\left(y_{1}\right)$: (1) is repulsive (>0) for short distances in $0\leq y_{1}\leq y_{1,0}$, (2) is attractive (<0) in $y_{1,0}\leq y_{1}\leq y_{1,1}$, (3) is repulsive in $y_{1,1}\leq y_{1}\leq y_{1,2}$, and (4) vanishes very quickly for $y_{1}>y_{1,2}$. The term $y_{1}$ varies inside the microscopically large but finite volume of each copy of the ensemble referred to at the beginning of this section. In short, $U_{1}\left(y_{1}\right)$ vanishes very quickly beyond a domain having a size of order of at most a few bond lengths in the te chain.

Another crucial interaction potential $U_{0}$, activation of the polymerization process, is supposed among the individual units, unit 2 in te, and the *cat*. The catalyst (also denoted here as unit 0) interacts simultaneously with the atomic unit (unit 1) in the ensemble and unit 2 in the te through the real, spherically symmetric potential $U_{0}\left(y_{0},y_{0}+y_{1}\right)$, with $y_{0}=R_{1}-R_{0}$. The properties of $U_{0}\left(y_{0},y_{0}+y_{1}\right)$ will not be discussed at this stage. Its assumed effective behavior (specifically, that of the average of $U_{0}\left(y_{0},y_{0}+y_{1}\right)$ over $y_{0}$) will be considered a posteriori in Section 4.3.

Units 0 and 1 will be included in the overall CM and in the general description in Section 3.2, where they are enlarged with $y_{0}$, $y_{1}$. The model is being constructed with the following crucial numbering convention. The set of all material entities is numbered successively in the following sequence: 0 (*cat*), 1 (individual unit), and 2,3,…$N_{te}$ (those in the te) consistently with the definitions and numbering of the y. In turn, such a numbering convention and the above choice of potentials $U_{1}\left(y_{1}\right)$ and $U_{0}\left(y_{0},y_{0}+y_{1}\right)$ will be consistent with the individual unit 1 to be bound to unit 2 in the te. Accordingly, we also introduce $A_{00}=M_{0}^{-1}+M_{1}^{-1}$, $A_{01}=A_{10}=-M_{1}^{-1}$ and so on for $A_{11}$, $A_{12}$, and $A_{21}$ upon consistently extending the definitions of $A_{ij}$ in Section 3.2.

The quantum purely kinetic Hamiltonian for the *cat*, the individual units, and the template is $\Pi_{3,0}^{2}/2M_{0}+\Pi_{1}^{2}/2M_{1}+P_{te,CM}^{2}/2M_{te}+2^{-1}\sum_{i,j=2}^{N_{te}-1}A_{ij}\left[-i\hbar \nabla_{y_{i}}\right]\left[-i\hbar \nabla_{y_{j}}\right]$.

At this stage, one performs the following transformations: (i) one introduces and factors out the contribution of the total center-of-mass (for te, unit 1, and *cat*); (ii) one introduces all relative vectors $y_{0}$, $y_{1}$, $y_{2}$,…, $y_{N_{te}-1}$ and makes use of Equation (74) for $y_{2}$,…, $y_{N_{te}-1}$; and (iii) one adds $U_{0}$, $U_{1}$, and all vibrational potentials accounting for the structure of the fj chain and implements the transition giving rise to fixed bond lengths in the te. Then, one infers the following effective quantum Hamiltonian (omitting the overall center-of-mass for all material entities): $\sum_{i,j=0}^{1}2^{-1}A_{ij}\left[-i\hbar \nabla_{y_{i}}\right]\left[-i\hbar \nabla_{y_{j}}\right]+\frac{A_{12}+A_{21}}{2d_{2}}e_{3,2}\left[-i\hbar \nabla_{y_{1}}\right]+\sum_{i,j=2}^{N_{te}-1}\frac{A_{ij}}{2d_{i}d_{j}}e_{3,i}e_{3,j}+U_{0}+U_{1}$.

It will be supposed that the *cat*, evolving in the fluid at the temperature *T* assumed, can be described approximately through classical statistical mechanics and that it has random motion during the effective duration of the process catalyzed by it. Let ${ß}_{3,c,0}$ be the classical momentum, which is canonically conjugate to $y_{0}$, for the classical *cat*.

As for the *cat*, the evolution of the fj te chain inside the fluid at thermal equilibrium at those temperatures is also described approximately through classical statistical mechanics: recall the analysis and the transition to classical variables in Section 3.2. Then, one can entertain the reasonableness of the following formal mixed quantum–classical Hamiltonian structure:

(78) $$\begin{array}{ccc} & & \frac{A_{00}{ß}_{3,c,0}^{2}}{2}+\frac{\left(A_{01}+A_{10}\right){ß}_{3,c,0}\left[-i\hbar \nabla_{y_{1}}\right]}{2}+2^{-1}A_{11}\left[-i\hbar \nabla_{y_{1}}\right]\left[-i\hbar \nabla_{y_{1}}\right]+\\ & & \frac{A_{12}+A_{21}}{2d_{2}}\left(-a_{3,c,2}\right)\left[-i\hbar \nabla_{y_{1}}\right]+\sum_{i,j=2}^{N_{te}-1}\frac{A_{ij}}{2d_{i}d_{j}}\left(-a_{3,c,i}\right)\left(-a_{3,c,j}\right)+U_{0}+U_{1} \end{array}$$

with the sole purpose of using it as a key guide to directly formulate a mixed quantum–classical Wigner–Liouville equation, as we shall do in Section 4.2.

### 4.2. Mixed Wigner–Liouville Equations for the Ensemble, te, and Catalyst

Let ${ß}_{3,c,1}$ be a momentum, which is canonically conjugate to $R_{1}$, for the individual unit treated quantum-mechanically. Use will be made of the classical variables employed in Section 3.2 and Section 4.1 for the te and catalyst, respectively.

The system formed by a unit, an fj chain, and a *cat* is described, by assumption, by a mixed (quantum–classical) distribution function in phase-space. The following quantum Wigner-like one for unit 1 and a classical Liouville-like one for the chain and the *cat* will be considered: $f_{m}=f_{m}\left(y_{1},\theta ,\varphi ,y_{0},{ß}_{3,c,1},\;\pi_{\theta ,c},\pi_{\varphi ,c},{ß}_{3,c,0};t\right)$. By assumption, $f_{m}$ fulfills the time (*t*)-reversible Wigner–Liouville equation that follows naturally by starting from the mixed Hamiltonian structure in (78) by operating quantum-mechanically (via Wigner) with it for the variables of the individual unit and classically (via Liouville) for those of the te and the catalyst. One finds directly:

(79) $$\begin{array}{ccc} & & \frac{\partial f_{m}}{\partial t}=-\left(A_{11}{ß}_{3,c,1}+A_{21}\left(-a_{3,c,2}\right)+A_{01}{ß}_{3,c,0}\right)\left(\nabla_{y_{1}}f_{m}\right)+\int d^{3}{ß}_{3,c,1,0}f_{m}\left({ß}_{3,c,1,0}\right)\int \frac{id^{3}y_{1,0}}{\hbar {\left(\pi \hbar \right)}^{3}}\times \\ & & \exp\left(\frac{2i\left({ß}_{3,c,1}-{ß}_{3,c,1,0}\right)y_{1,0}}{\hbar }\right)[U_{1}\left(y_{1}+y_{1,0}\right)-U_{1}\left(y_{1}-y_{1,0}\right)+\\ & & U_{0}\left(y_{0},y_{0}+y_{1}+y_{1,0}\right)-U_{0}\left(y_{0},y_{0}+y_{1}-y_{1,0}\right)]+\\ & & +A_{12}{ß}_{3,c,1}[\frac{\partial (-a_{3,c,2})}{\partial \theta_{2}}\frac{\partial f_{m}}{\partial \pi_{\theta_{2}}}-\\ & & \frac{\partial (-a_{3,c,2})}{\partial \pi_{\theta_{2}}}\frac{\partial f_{m}}{\partial \theta_{2}}+\frac{\partial (-a_{3,c,2})}{\partial \varphi_{2}}\frac{\partial f_{m}}{\partial \pi_{\varphi_{2}}}-\frac{\partial (-a_{3,c,2})}{\partial \pi_{\varphi_{2}}}\frac{\partial f_{m}}{\partial \varphi_{2}}]+{\left[{\tilde{H}}_{3,fj,c},f_{m}\right]}_{Pb}-\\ & & \left(A_{00}{ß}_{3,c,0}+A_{10}{ß}_{3,c,1}\right)\left(\nabla_{y_{0}}f_{m}\right)+\left(\nabla_{y_{0}}U_{0}\left(y_{0},y_{0}+y_{1}\right)\right)\left(\nabla_{{ß}_{3,c,0}}f_{m}\right), \end{array}$$

with ${\tilde{H}}_{3,fj,c}=\sum_{i,j=2}^{N-1}\left(A_{ij}/2d_{i}d_{j}\right)\left(-a_{3,c,i}\right)\left(-a_{3,c,j}\right)$ (recall Section 3.2). ${\left[{\tilde{H}}_{3,fj,c},f_{m}\right]}_{Pb}$ denotes the standard classical Poisson bracket [57] (recall Equation (77)). It approximates, in the classical regime, an integral contribution for the chain analogous to the one for the individual unit in Equation (79). We have interpreted the formulation of this mixed quantum–classical formulation by writing directly the pair $A_{01}{ß}_{3,c,0}\left(\nabla_{y_{1}}f_{m}\right)$ and $A_{10}{ß}_{3,c,1}\left(\nabla_{y_{0}}f_{m}\right)$ together with the pair $A_{21}\left(-a_{3,c,2}\right)\left(\nabla_{y_{1}}f_{m}\right)$ and $A_{12}{ß}_{3,c,1}$ times (the Poisson bracket of $\left(-a_{3,c,2}\right)$ and $f_{m}$). Instead of the mixed quantum–classical Equation (79), a more basic treatment would have taken a fully quantum-mechanical Wigner equation for all (individual unit, te, and *cat*) entities, with Poisson brackets replaced by the corresponding integrals, as a starting point. However, in order not to encumber the analysis, it seemed more economical not to proceed like that but to start out from the mixed quantum–classical Equation (79). We advance that both te and *cat* will be supposed to be in classical states at approximate thermal equilibrium in the next subsection, which also supports such a shortened strategy. The term $f_{m}\left({ß}_{3,c,1,0}\right)$ inside the integral in Equation (79) is (omitting the writing of repeated variables) obtained just by replacing ${ß}_{3,c,1}$ with ${ß}_{3,c,1,0}$ in $f_{m}$.

Through direct partial integrations, total probability is shown to be conserved consistently: $\partial \int d^{3}y_{1}\int d^{3}{ß}_{3,c,1}\int d^{3}y_{0}\int d^{3}{ß}_{3,c,0}\int {\left[d\Omega \right]}_{3,L}f_{m}/\partial t=0$.

### 4.3. Template and Catalyst at Thermal Equilibrium: Integration over Their Degrees of Freedom and Non-Standard Effective Hamiltonian

The dynamics will be considered for a sufficiently long time (*t*). As the fluid is at thermal equilibrium at absolute temperature *T*, it seems physically reasonable that in each triplet, the te chain and *cat* are approximately at thermal equilibrium at the same *T*, and that under their influence, the individual atomic unit evolves off-equilibrium so as to give rise to polymerization. The resulting dynamics of the individual atomic unit are not expected to alter the statistical equilibrium states of the fj chain and of the *cat*. Actually, such an assumption underlies the very formulation of Equation (79).

We accept the approximate factorization of the non-equilibrium distribution: $f_{m}≃f_{1}f_{eq,2}f_{eq,0}$ with an off-equilibrium $f_{1}=f_{1}\left(y_{1},{ß}_{3,c,1},t\right)$ for the individual atomic unit. The *cat* is described by the (*t*-independent) classical Boltzmann equilibrium distribution ($A_{10}=A_{01}$):

$f_{eq,0}=Z_{eq,0}^{-1}\exp\left[-{\left(K_{B}T\right)}^{-1}\left[\left(A_{00}/2\right){ß}_{3,c,0}^{2}+A_{10}{ß}_{3,c,0}{ß}_{3,c,1}+U_{0}\right]\right]$, which depends on ${ß}_{3,c,0}$, ${ß}_{3,c,1}$, $y_{0}$, and $y_{1}$ (with $Z_{eq,0}=\int d^{3}y_{0}d^{3}{ß}_{3,c,0}\exp\left[-{\left(K_{B}T\right)}^{-1}\left[\left(A_{00}/2\right){ß}_{3,c,0}^{2}+U_{0}\right)\right]]$).

We also accept that the te chain is described by the (*t*-independent) classical Boltzmann equilibrium distribution ($A_{12}=A_{21}$): $f_{eq,2}=Z_{eq,2}^{-1}\exp\left[-{\left(K_{B}T\right)}^{-1}\left[\left(A_{12}/d_{2}\right){ß}_{3,c,1}\left(-a_{3,c,2}\right)+{\tilde{H}}_{3,fj,c,2}\right]\right]$, which depends on all variables of the te and ${ß}_{3,c,1}$ (with $Z_{eq,2}={\left[d\Omega \right]}_{3,L}\exp\left[-{\left(K_{B}T\right)}^{-1}{\tilde{H}}_{3,fj,c,2}\right]$), which includes ${ß}_{3,c,1}\left(-a_{3,c,2}\right)$.

Notice that $\left(-a_{3,c,2}\right)$ is coupled to $\nabla_{y_{1}}$ in Equation (79).

We integrate Equation (79) with $\int {\left[d\Omega \right]}_{3,L}\int d^{3}y_{0}d^{3}{ß}_{3,c,0}$ and perform the approximate replacement $f_{m}≃f_{1}f_{eq,2}f_{eq,0}$

The contribution of $A_{12}{ß}_{3,c,1}$ times the Poisson bracket involving $f_{1}f_{eq,2}f_{eq,0}$ and multiplying it plus the contribution of the Poisson bracket (${\left[{\tilde{H}}_{3,fj,c,2},f_{1}f_{eq,2}f_{eq,0}\right]}_{Pb}$) gives a vanishing result.

Then: $\int {\left[d\Omega \right]}_{3,L}f_{m}≃f_{1}f_{eq,0}\int {\left[d\Omega \right]}_{3,L}f_{eq,2}$, and $\int {\left[d\Omega \right]}_{3,L}f_{m}\left(-a_{3,c,2}\right)≃f_{1}f_{eq,0}\int {\left[d\Omega \right]}_{3,L}f_{eq,2}\left(-a_{3,c,2}\right)=f_{1}f_{eq,0}\left[-\left(\left(K_{B}Td_{2}\right)/A_{12}\right)\left(\nabla_{{ß}_{3,c,1}}\int {\left[d\Omega \right]}_{3,L}f_{eq,2}\right)\right]$.

The function $\int {\left[d\Omega \right]}_{3,L}f_{eq,2}\equiv f_{2}=f_{2}\left({ß}_{3,c,1}\right)$ is studied in the Appendix A.

Consequently: $\int d^{3}y_{0}d^{3}{ß}_{3,c,0}\int {\left[d\Omega \right]}_{3,L}f_{m}≃f_{1}f_{2}f_{0}$, with

$f_{0}=\int d^{3}y_{0}d^{3}{ß}_{3,c,0}f_{eq,0}=\exp\left[{\left(2K_{B}T\right)}^{-1}\left(A_{10}^{2}/A_{00}\right){ß}_{3,c,1}^{2}\right]$.

Equation (79) becomes, in terms of $W=W\left(y_{1},{ß}_{3,c,1},t\right)=f_{1}f_{2}f_{0}$:

(80) $$\begin{array}{ccc} & & \frac{\partial W}{\partial t}=-\left[A_{11}{ß}_{3,c,1}-\left(K_{B}T\right)\left(\nabla_{{ß}_{3,c,1}}\ln f_{2}\right)-\left(A_{10}^{2}/A_{00}\right){ß}_{3,c,1}\right]\left(\nabla_{y_{1}}W\right)+\int d^{3}{ß}_{3,c,1,0}\times \\ & & W\left(y_{1},{ß}_{3,c,1,0},t\right)]\int \frac{id^{3}y_{1,0}}{\hbar {\left(\pi \hbar \right)}^{3}}\exp\left(\frac{2i\left({ß}_{3,c,1}-{ß}_{3,c,1,0}\right)y_{1,0}}{\hbar }\right)[U_{1,eff}\left(|y_{1}+y_{1,0}|\right)-\\ & & U_{1,eff}\left(|y_{1}-y_{1,0}|\right)] \end{array}$$

(81) $$\begin{array}{ccc} & & U_{1,eff}\left(|y_{1}|\right)=U_{1}\left(|y_{1}|\right)+U_{0,eff}\left(|y_{1}|\right) \end{array}$$

(82) $$\begin{array}{ccc} & & U_{0,eff}\left(|y_{1}|\right)=\frac{\int d^{3}y_{0}U_{0}\left(y_{0},y_{0}+y_{1}\right)\exp\left[-{\left(K_{B}T\right)}^{-1}U_{0}\right)]}{\int d^{3}y_{0}\exp\left[-{\left(K_{B}T\right)}^{-1}U_{0}\right]} \end{array}$$

We shall assume the following properties of $U_{0,eff}\left(|y_{1}|\right)=U_{0,eff}\left(y_{1}\right)$ ($y_{1}=|y_{1}|$): ($a_{1}$) it is repulsive for $0\leq y_{1}\leq y_{1,3}$, where $y_{1,0}\leq y_{1,3}<y_{1,1}$; ($a_{2}$) it is attractive for $y_{1,3}\leq y_{1}\leq y_{1,2}$; and ($a_{3}$) it tends to vanish for adequately large values of $y_{1}\left(>y_{1,2}\right)$. We allow, at this stage, for $U_{0}\left(y_{0},y_{0}+y_{1}\right)$ to give rise to bound states of the catalyst for the system formed by the individual unit and the te. Such a possibility can be entertained at the level of Equation (79) but lies outside the scope of Equation (80) and its consequences, which concentrate on the individual atom 1. See the comments in Section 5.

Upon recalling the properties assumed for $U_{1}$, it follows that $U_{1,eff}\left(|y_{1}|\right)=U_{1,eff}\left(y_{1}\right)$: (1) is repulsive in $0\leq y_{1}\leq y_{1,0}$; (2) is attractive in $y_{1,0}\leq y_{1}\leq y_{1,1}$; (3) is repulsive in $y_{1,1}\leq y_{1}\leq y_{1,2}$; and (4) tends to vanish for adequately large values of $y_{1}\left(>y_{1,2}\right)$. Two important additional points are: (5) $U_{1,eff}\left(y_{1}\right)$ continues to be attractive in $y_{1,0}\leq y_{1}\leq y_{1,1}$ in spite of the possibility that $U_{0,eff}\left(y_{1}\right)$ can be repulsive in $y_{1,0}\leq y_{1}\leq y_{1,3}$; and (6) $U_{1,eff}\left(y_{1}\right)$ is considerably less repulsive than $U_{1}\left(y_{1}\right)$ in $y_{1,1}\leq y_{1}\leq y_{1,2}$. The basic effect due to $U_{0,eff}\left(y_{1}\right)$ (and, hence, due to the catalyst) is to offset and make lower the positive values of $U_{1}\left(y_{1}\right)$ in $y_{1,1}\leq y_{1}\leq y_{1,2}$. We shall assume later (Section 4.5) that $U_{1,eff}\left(y_{1}\right)$ does give rise to bound states (specifically, to one bound state).

Equation (80) depends only on the degrees of freedom of the individual unit. Equation (80) with (81) is the standard Wigner equation for the non-standard quantum Hamiltonian: ${\tilde{H}}_{n-s,1}=-\left(\hbar^{2}/2\right)\left(\left(A_{11}-\left(A_{10}^{2}/A_{00}\right)\right)\right)\nabla_{y_{1}}^{2}+\left[\ln f_{2}\right]\left({ß}_{3,c,1}\to -i\hbar \nabla_{y_{1}}\right)+U_{1,eff}\left(|y_{1}|\right)$. In so doing, we are correcting some misprint in the non-standard quantum Hamiltonian ${\tilde{H}}_{n-s,1}$ in Section 5.1 in [33]: such a misprint is inconsequential regarding the developments in [33].

Equation (80) directly yields the probability flux conservation:

(83) $$\begin{array}{ccc} & & \frac{\partial }{\partial t}\int d^{3}{ß}_{3,c,1}W=-\nabla_{y_{1}}\int d^{3}{ß}_{3,c,1}[A_{11}{ß}_{3,c,1}-\left(K_{B}T\right)\left(\nabla_{{ß}_{3,c,1}}\ln f_{2}\right)-\\ & & -\left(A_{10}^{2}/A_{00}\right){ß}_{3,c,1}]W \end{array}$$

(84) $$\begin{array}{ccc} & & \frac{\partial }{\partial t}\int d^{3}y_{1}\int d^{3}{ß}_{3,c,1}W=0 \end{array}$$

### 4.4. Standard Approximate Effective Quantum Hamiltonian for the Individual Unit

As it is difficult to handle ${\tilde{H}}_{n-s,1}$, it will be approximated by the new effective Hamiltonian ${\tilde{H}}_{eff,1}$ below. Accordingly, one approximates: $-\left(K_{B}T\right)\left(\nabla_{{ß}_{3,c,1}}\ln f_{2}\right)≃A_{12}\sigma {ß}_{3,c,1}$: see Appendix A. The constant σ(>0 and dimensionless) accounts for the influence of the classical fj chain on the dynamics of unit 1. After this approximation, Equation (80) becomes the standard Wigner equation for the effective standard quantum Hamiltonian for unit 1: ${\tilde{H}}_{eff,1}=-\left(\hbar^{2}/2\right)A_{11,eff}\nabla_{y_{1}}^{2}+U_{1,eff}\left(|y_{1}|\right)$, where $A_{11,eff}=\left(A_{11}-\left(A_{10}^{2}/A_{00}\right)\right)+A_{12}\sigma$, which yields a (quasi-)continuous spectrum of eigenvalues and one bound state associated with (quasi-)unbound motion and polymerization of the individual unit, respectively.

Let $W=W(y_{1},{ß}_{3,c,1},t)$ be the (effective) non-equilibrium Wigner function for a quantum particle with mass $A_{11,eff}^{-1}$ and subject to the potential $U_{1,eff}\left(|y_{1}|\right)$. The corresponding non-equilibrium Wigner equation reads:

(85) $$\begin{array}{ccc} & & \frac{\partial W}{\partial t}=-\left[A_{11,eff}{ß}_{3,c,1}\right]\left(\nabla_{y_{1}}W\right)+\int d^{3}{ß}_{3,c,1,0}W\left(y_{1},{ß}_{3,c,1,0},t\right)]\times \\ & & \int \frac{id^{3}y_{1,0}}{\hbar {\left(\pi \hbar \right)}^{3}}\exp\left(\frac{2i\left({ß}_{3,c,1}-{ß}_{3,c,1,0}\right)y_{1,0}}{\hbar }\right)\left[U_{1,eff}\left(|y_{1}+y_{1,0}|\right)-U_{1,eff}\left(|y_{1}-y_{1,0}|\right)\right] \end{array}$$

The equilibrium distribution determined by Equation (85) is $W_{eq}$.

### 4.5. Extension of Section 2.8 and Section 2.9 to Equation (85): Small Thermal Wavelength and Long-Time Approximations

The one-dimensional analysis in Section 2 can now be directly extended to the D=3 Equation (85): namely, equilibrium distribution $W_{eq}$, the family of orthogonal polynomials generated by the former, non-equilibrium moments and hierarchy, and small thermal wavelength (corresponding to the absolute temperature *T* assumed for the fluid) and long-time approximations. Details will be omitted. Then, the 1D long-time approximations in Section 2.9, extended directly to 3D by following [30], lead to the irreversible D=3 Smoluchowski equation for the lowest moment $W_{\left[0\right]}=W_{\left[0\right]}\left(y_{1},t\right)\left(=\int d^{3}{ß}_{3,c,1}\right)W\left(y_{1},{ß}_{3,c,1},t\right))$ for the individual atomic unit ($y_{1}=\left(y_{1,1},y_{1,2},y_{1,3}\right)$):

(86) $$\begin{array}{ccc} & & \frac{\partial W_{\left[0\right]}}{\partial t}=Dq_{eq}A_{11,eff}\sum_{\alpha =1}^{3}\frac{\partial }{\partial y_{1,\alpha }}M_{\left[1_{\alpha }\right],\left[0\right]}W_{\left[0\right]} \end{array}$$

(87) $$\begin{array}{ccc} & & M_{\left[1_{\alpha }\right],\left[0\right]}W_{\left[0\right]}=-q_{eq}A_{11,eff}\frac{\partial }{\partial y_{1,\alpha }}\left(\epsilon_{\left[2],[0\right]}W_{\left[0\right]}\right)++\frac{1}{q_{eq}}\frac{\partial U_{1,eff}}{\partial y_{1,\alpha }}W_{\left[0\right]} \end{array}$$

*D* (assumed to be $y_{1}$-independent ) and $\epsilon_{\left[2],[0\right]}$ are the natural 3D counterparts of the 1D ones in Section 2.9. The equilibrium distribution for (87) is:

$W_{[0],eq}=\sum_{j}\exp\left(-E_{j}/\left(K_{B}T\right)\right)\phi_{j}{\left(y_{1}\right)}^{*}\phi_{j}\left(y_{1}\right)$.

The terms $\phi_{j}\left(y_{1}\right)$ and $E_{j}$ are, for all possible values of the set of subindices *j*, the (almost) continuum and bound-state eigenfunctions and energies of ${\tilde{H}}_{eff,1}$ (${\tilde{H}}_{eff,1}\phi_{j}\left(y_{1}\right)=E_{j}\phi_{j}\left(y_{1}\right)$). Since $U_{1,eff}=U_{1,eff}\left(|y_{1}|\right)=U_{1,eff}\left(y_{1}\right)$, $W_{eq}$ is seen to depend on $y_{1}^{2}$, ${ß}_{3,c,1}^{2}$, and ${\left(y_{1}{ß}_{3,c,1}\right)}^{2}$. Then: $\epsilon_{\left[2],[0\right]}=-\int d^{3}{ß}_{3,c,1}W_{eq}{\left({ß}_{3,c,1}\right)}_{\alpha }^{2}/\left(q_{eq}^{2}\int d^{3}{ß}_{3,c,1}W_{eq}\right)\left(<0\right)$ is seen to be independent on α=1,2,3 and to depend only on $y_{1}$. Consistently, $W_{[0],eq}$ fulfills: $M_{\left[1_{\alpha }\right],\left[0\right]}W_{[0],eq}=0$, which is a set of three partial differential equations that are compatible and explicitly solvable for $W_{[0],eq}$, since $U_{1,eff}$ and $\epsilon_{\left[2],[0\right]}$ depend on $y_{1}$. We shall assume that $U_{1,eff}$ gives rise to just one bound state.

The physically interesting solution is, naturally, spherically symmetric $W_{\left[0\right]}=W_{\left[0\right]}\left(y_{1},t\right)$ so that Equation (86) becomes:

(88) $$\begin{array}{ccc} & & \frac{\partial W_{\left[0\right]}}{\partial t}=Dq_{eq}A_{11,eff}\left(\frac{\partial }{\partial y_{1}}+\frac{2}{y_{1}}\right)\left(-q_{eq}A_{11,eff}\frac{\partial }{\partial y_{1}}\left(\epsilon_{\left[2],[0\right]}W_{\left[0\right]}\right)+\frac{1}{q_{eq}}\frac{\partial U_{1,eff}}{\partial y_{1}}W_{\left[0\right]}\right) \end{array}$$

now with $q_{eq}={\left(2K_{B}T/A_{11,eff}\right)}^{1/2}$. It is convenient to replace $W_{\left[0\right]}$ with another distribution $f=f\left(y_{1},t\right)=y_{1}^{-2}W_{\left[0\right]}\left(y_{1},t\right)$. Equation (88) becomes:

(89) $$\begin{array}{ccc} & & \frac{\partial f}{\partial t}=Dq_{eq}A_{11,eff}\frac{\partial }{\partial y_{1}}(-q_{eq}A_{11,eff}\frac{\partial }{\partial y_{1}}\left(\epsilon_{\left[2],[0\right]}f\right)+\frac{1}{q_{eq}}\frac{\partial U_{1,eff}}{\partial y_{1}}f+\\ & & \frac{2}{y_{1}}q_{eq}\epsilon_{\left[2],[0\right]}A_{11,eff}f \end{array}$$

### 4.6. Mean First Passage Time

It is important to compute approximately the (average) time required for unit 1 to become attached to the chain as a next-neighbor of unit 2 in the te chain in the presence of and under the influence of the *cat*. For that purpose, the mean first passage time (MFPT) formalism, which provides an estimate of the latter time, is very useful. For references about the MFPT formalism, see [18,47,58]. In the present application of the MFPT, we shall extend [30,33,59]. The MFPT $t(y_{1})$ function is the solution of the following so-called adjoint equation associated with Equation (89):

(90) $$\begin{array}{ccc} & & Dq_{eq}^{2}A_{11,eff}^{2}\left(-\epsilon_{\left[2],[0\right]}\right)\frac{\partial^{2}t\left(y_{1}\right)}{\partial y_{1}^{2}}-Dq_{eq}A_{11,eff}(\frac{1}{q_{eq}}\frac{\partial U_{1,eff}}{\partial y_{1}}+\\ & & \frac{2q_{eq}\epsilon_{\left[2],[0\right]}A_{11,eff}}{y_{1}})\frac{\partial t(y_{1})}{\partial y_{1}}=-1 \end{array}$$

provided that suitable boundary conditions are added. The properties of $U_{1,eff}\left(y_{1}\right)$ have been explained in Section 4.3. Accordingly, the boundary conditions adequate for polymerization are the following: $t(y_{abs})=0$ (absorption) and ${\left[\partial t\left(y_{1}\right)/\partial y_{1}\right]}_{y_{1}=y_{ref}}=0$ (reflection). The radial distance $y_{ref}$ is supposed to fulfill: $y_{1,0}\leq y_{ref}\leq y_{1,1}$. The radial distance $y_{abs}$ is supposed to be larger than (but close to) $y_{1,2}$. The term $t(y_{1})$ is interpreted here as an estimate of the time required for the individual atom 1—near $y_{abs}$ and, thus, far from unit 2 of the te—to reach $y_{1}$ under the action of the *cat*. If $y_{1,0}\leq y_{1}\leq y_{1,1}$, then $t(y_{1})$ estimates the time required for unit 1 to become bound to unit 2 of the te.

Then, by direct integration, the solution of Equation (90) with those boundary conditions is:

(91) $$\begin{array}{ccc} & & t\left(y_{1}\right)=\int_{y_{1}}^{y_{abs}}\frac{ds_{1}}{s_{1}^{2}D{\left(q_{eq}A_{11,eff}\right)}^{2}}J\left(s_{1}\right) \end{array}$$

(92) $$\begin{array}{ccc} & & J\left(s_{1}\right)=-\int_{y_{ref}}^{s_{1}}\frac{ds_{2}s_{2}^{2}}{\epsilon_{\left[2],[0\right]}\left(s_{2}\right)}\exp\left[-\frac{1}{2K_{B}T}\int_{s_{2}}^{s_{1}}\frac{ds_{3}\left(\partial U_{1,eff}/\partial s_{3}\right)}{\epsilon_{\left[2],[0\right]}\left(s_{3}\right)}\right] \end{array}$$

For definiteness, we have chosen $y_{1,2}=y_{abs}$ in Equation (91).

Reference [30] studied an MFPT for a simpler chemical reaction between two atoms with neither a te molecular chain nor a *cat* (and not having required to start out from any sort of mixed Liouville–Wigner equation, as has been the case here). In spite of that relative simplicity, the resulting MFPT is rather similar to that described by Equations (91) and (92). The detailed analysis of the MFPT in [30] can be extended rather directly to Equations (91) and (92) and will be omitted. The resulting MFPT in [30] displays a temperature dependence consistent with the Arrhenius formula for rate constants in chemical reactions, and the same is true for Equations (91) and (92). For brevity, we shall limit ourselves to a direct estimate based upon the properties of $U_{1,eff}$: yielding the Arrhenius formula here.

We suppose that $y_{1}$ fulfills $y_{ref}\leq y_{1}\leq y_{1,1}$. We choose suitable estimates for $-\epsilon_{\left[2],[0\right]}$ to let them to be displaced without large errors outside $\int_{s_{2}}^{s_{1}}ds_{3}$, and we subsequently perform the resulting integration $\int_{s_{2}}^{s_{1}}ds_{3}\left(\partial U_{1,eff}/\partial s_{3}\right)$. Then, we argue that an estimate of the dominant contribution to the exponential inside $J(s_{1})$ is: $\exp[\frac{1}{2K_{B}T}\left[\frac{U_{1,eff}}{\left(-\epsilon_{\left[2],[0\right]}\right)}\left(y_{+}\right)-\frac{U_{1,eff}}{\left(-\epsilon_{\left[2],[0\right]}\right)}\left(y_{-}\right)\right]]$. The radial distance $y_{+}$ is larger than $y_{1,1}$ and is not far from the $y_{1}$ at which $\frac{U_{1,eff}}{\left(-\epsilon_{\left[2],[0\right]}\right)}$ is positive (with repulsive $U_{1,eff}$) and takes on its largest values. The radial distance $y_{-}$ is smaller than $y_{1,1}$ and is not far from the $y_{1}$ at which $\frac{U_{1,eff}}{\left(-\epsilon_{\left[2],[0\right]}\right)}$ is negative (with attractive $U_{1,eff}$) and takes on its minimum values. We remind that $-\epsilon_{\left[2],[0\right]}$ is positive (Section 2.7). It is plausible that $-\epsilon_{\left[2],[0\right]}\left(y_{+}\right)$ ($-\epsilon_{\left[2],[0\right]}\left(y_{-}\right)$) is dominated by the almost continuous spectrum (the bound state). Then, an estimate of the the MFPT is:

(93) $$\begin{array}{ccc} & & t\left(y_{1}\right)≃\int_{y_{1}}^{y_{abs}}\frac{ds_{1}}{s_{1}^{2}D{\left(q_{eq}A_{11,eff}\right)}^{2}}\int_{y_{ref}}^{s_{1}}\frac{ds_{2}s_{2}^{2}}{\left(-\epsilon_{\left[2],[0\right]}\left(s_{2}\right)\right)}\times \\ & & \exp[\frac{1}{2K_{B}T}\left[\frac{U_{1,eff}}{\left(-\epsilon_{\left[2],[0\right]}\right)}\left(y_{+}\right)-\frac{U_{1,eff}}{\left(-\epsilon_{\left[2],[0\right]}\right)}\left(y_{-}\right)\right]] \end{array}$$

Other contributions to $J(s_{1})$ are regarded as subdominant and, hence, discarded. The term $t{\left(y_{1}\right)}^{-1}$ for $y_{ref}\leq y_{1}\leq y_{1,1}$ provides an approximate estimate of a rate constant for polymerization activated by the *cat*. Then, notice that factor $\exp[-\frac{1}{2K_{B}T}\left[\frac{U_{1,eff}}{-\epsilon_{\left[2],[0\right]}}\left(y_{+}\right)-\frac{U_{1,eff}}{-\epsilon_{\left[2],[0\right]}}\left(y_{-}\right)\right]]$ is responsible for and characteristic of an Arrhenius behavior. It also explains why the inclusion of the *cat* activates polymerization: in fact, the latter (due to $U_{0,eff}<0$) makes $U_{1,eff}\left(>0\right)$ be smaller than $U_{1}\left(>0\right)$ in $y_{1,1}\leq y_{1}\leq y_{1,2}$.

One can obtain directly another solution of Equation (90) with other boundary conditions: namely, with reflection for $y_{1}\geq y_{1,2}$ and reflection for $y_{1}\leq y_{1,1}$. However, such a solution has been discarded as it implies properties that disagree with the physically expected ones (implied correctly by Equations (91) and (92)).

## 5. Conclusions and Discussion

The first part (Section 2) of this work dealt with the basic quantum Wigner function and non-equilibrium equation for a microscopic particle subject to a potential *V* and to a heat bath (HB) at thermal equilibrium. Previous analyses were extended non-trivially. For simplicity, only 1D was considered, with the extension to 3D being direct. The case in which *V* has one bound state (plus an infinite number of discrete states that approximate the standard continuum or scattering states) was considered. The equilibrium Wigner distribution generates an infinite number of orthogonal polynomials in momentum. The latter enabled us to define an infinite family of non-equilibrium moments. Commonly, Wigner functions are employed to evaluate expectation values for suitable operators in phase space (see, for instance [7,8]). On the other hand, the present approach is not specifically concerned with phase space; rather, the former focuses on the information encoded in non-equilibrium moments (depending on the spatial positions of the particles as time evolves and becomes long): specifically, in the lowest moment, which can give probabilistic information. The non-equilibrium Wigner equation yields a general *n*-term hierarchy for the corresponding moments. A new non-trivial solution of the non-equilibrium hierarchy that combines operator-continued fractions and the infinite series thereof is obtained and analyzed; arguments are given to support its finiteness. In a short thermal wavelength regime (retaining quantum features and adequate for chemical reactions), the non-equilibrium hierarchy is approximated by a three-term one. In a long-time approximation, the approximate three-term hierarchy is, in turn, approximated by a Smoluchovski equation. Among other open issues left open by the present study, we quote here the following: (i) further improvements regarding the theory of the generalized orthogonal polynomials in [41], which can benefit the construction of the $H_{Q,n}\left(y\right)$; (ii) further analysis for infinite *n*-term hierarchies (n>3); and (iii) mathematically rigorous analysis of continued fractions of operators. In fact, in previous works (see [29,30] and references therein) and in the present one, we have handled continued fractions of operators and the infinite series thereof in a formal way, with effort to provide arguments to justify consistency, convergence, and an approximate approach to equilibrium for long *t*. Recall, for instance, the operator *A* in Section 2.6. However, regarding (iii) in particular, we have been recognizedly unable to obtain mathematically rigorous results that, to the best of our knowledge, appear to be lacking.

In the second part (Section 3 and Section 4) of this work, a new model of the growth (polymerization) of a molecular chain (template, te) by binding an individual atom activated by a catalyst is developed in 3D. The atom, te, and *cat* move randomly as solutions in a fluid at rest (playing the role of an HB) in thermal equilibrium. Classical statistical mechanics describe te and *cat* approximately. The individual atom is treated quantum-mechanically. Mixed non-equilibrium quantum–classical Wigner–Liouville functions and dynamical equations for the individual atom and for the te and *cat*, respectively, are employed. By assuming the latter two to be at thermal equilibrium, integrating over their degrees of freedom, and through a further approximation regarding the degrees of freedom of the te, a standard 3D effective non-equilibrium Wigner equation is obtained for the individual atom. Upon extending to the latter Wigner equation the moment methods together with the short thermal wavelength and long-time approximations in Section 2.8 and Section 2.9, respectively, an approximate 3D Smoluchowski equation is obtained for the individual atom. The mean first passage time (MFPT) for the individual atom to become bound to the te, facilitated by the *cat*, is considered. The resulting MFPT displays a temperature dependence consistent with the Arrhenius formula for rate constants in chemical reactions.

The following properties of typical insertion polymerizations occurring in practice should be mentioned (see Section 3.6.1 in [32]). Firstly, the *cat* does not emerge unchanged as a consequence of the polymerization process: that is, its final state is different from the initial one. Secondly, it continues to reside, in the form of deteriorated fragments, at the relevant end of the augmented te chain.

**Figure 1:**
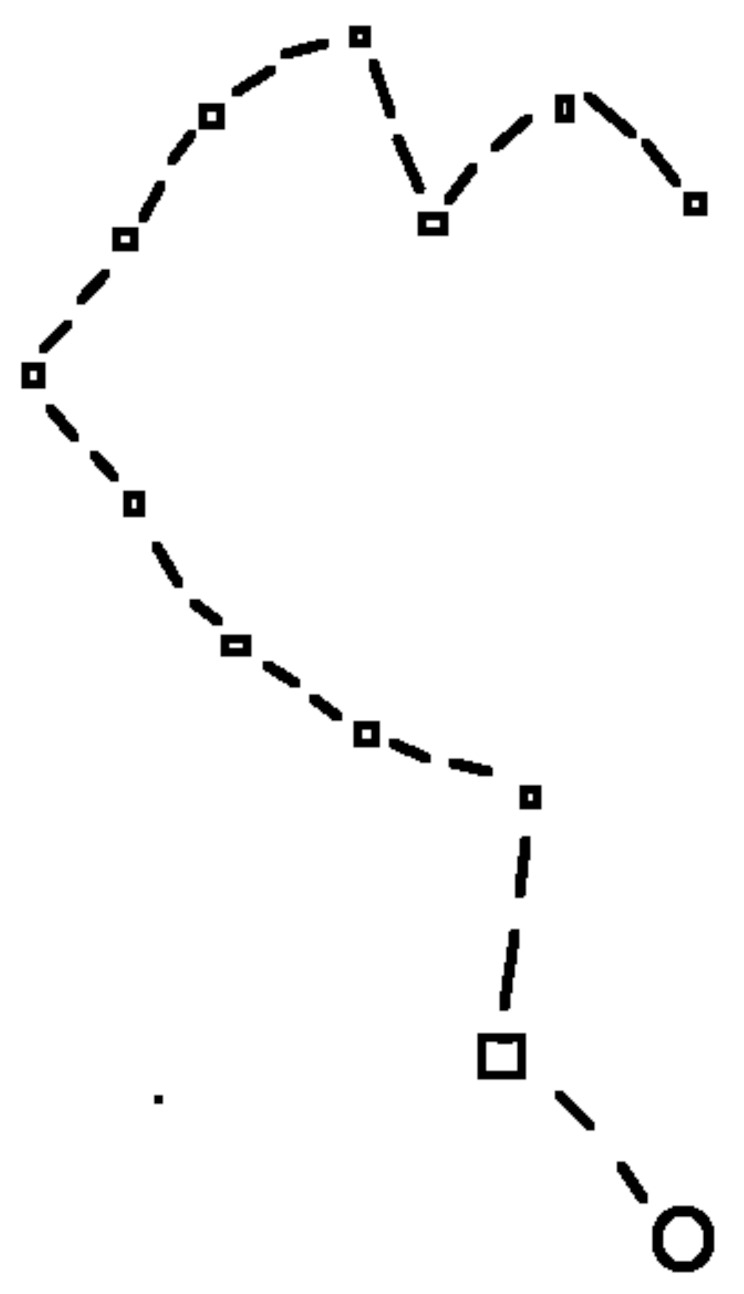
Catalyst (circle), individual unit atom (box), and freely jointed template formed by 11 units (small boxes). Dashed lines represent binding (bonds). The successive numbering is: catalyst (unit 0), individual atom 1, and, in the template, atom 2, atom 3,…, atom 11. After polymerization, atom 1 becomes bound to atom 2, and the catalyst is bound (weakly) to atoms 1 and 2. The term $y_{1}$ is the vector (not displayed) along the dashed line from atom 1 (box) to atom 2 (small box).

Motivated by those facts, two comments regarding the model presented here seem in order. Our treatment allows for the catalyst to remain weakly bound to the enlarged chain formed by atom 1 bound to the initial te chain: that can, in principle, occur for suitable $U_{0}\left(y_{0},y_{0}+y_{1}\right)$. The model, with the chosen numbering of material units and dynamical variables, has been formulated precisely in order to account for that: the individual unit is numbered atom 1 and will become bound to atom 2 (one end atom in the te). Then, at the end of the process, the *cat* (numbered as 0) may remain bound to atom 1 as a possible additional extension of the chain (see Figure 1). On the other hand, our treatment does not seem to account for the deterioration of the catalyst: a generalization (outside our scope here) would be required for that.

## Data Availability

Data are contained within the article.

## Appendix A. 3D Single-Unit Polymerization by Classical fj Chain at Equilibrium: Computations

The formula $f_{2}=f_{2}\left({ß}_{3,c,1}\right)=\int {\left[d\Omega \right]}_{3,L}f_{eq,2}$ will be studied here by extending [33]. First, Gaussian integrations over $\Pi_{i=2}^{N_{te}-1}d\varphi_{i}d\pi_{\varphi ,c,i}$ in ${\left[d\Omega \right]}_{3,L}$ are performed by generalizing directly the rotational invariant methods in [60]. The result is:

(A1) $$\begin{array}{ccc} & & f_{2}=\exp\left[\frac{A_{12}^{2}{\left(A_{2}^{-1}\right)}_{22}{ß}_{3,c,1}^{2}}{2K_{B}T}\right]{\left({\left[\int (\frac{{\left[d\Omega \right]}_{3,2}}{det\Delta_{2}}\right)}^{1/2}\right]}^{-1}\int (\frac{{\left[d\Omega \right]}_{3,2}}{det\Delta_{2}}{)}^{1/2}\times \\ & & \exp[-\frac{A_{12}}{2K_{B}T}\sum_{i,j=2}^{N_{te}-1}\left(A_{12}{\left(A_{2}^{-1}\right)}_{2i}\right)\left({ß}_{3,c,1}u_{i}\right){\left({\left(\Delta_{2,1}\right)}^{-1}\right)}_{ij}\left(A_{12}{\left(A_{2}^{-1}\right)}_{j2})\left({ß}_{3,c,1}u_{j}\right)\right] \end{array}$$

The $\left(N_{te}-2\right)\times \left(N_{te}-2\right)$ matrix $A_{2}$ with non-vanishing elements $A_{ij}$, $i,j=2,\ldots ,N_{te}-1$ is symmetric, tridiagonal, and has positive eigenvalues. $A_{2}^{-1}$ and $detA_{2}$ are the inverse and the determinant, respectively, of $A_{2}$. The $\left(N_{te}-2\right)\times \left(N_{te}-2\right)$ matrices $\Delta_{2}$ and $\Delta_{2,1}$ have elements: ${\left(\Delta_{2}\right)}_{ij}={\left(A_{2}^{-1}\right)}_{ij}u_{i}u_{j}$ and ${\left(\Delta_{2,1}\right)}_{ij}=A_{12}{\left(A_{2}^{-1}\right)}_{ij}u_{i}u_{j}$, respectively. The integral in Equation (A1) with ${\left(\Delta_{2,1}\right)}^{-1}=0$ has been studied in [53,60]: it was found that the dominant contributions are equal to one another and come from all tiny domains with ${\left(u_{i}u_{j}\right)}^{2}$ close to +1. One finds:

(A2) $$\begin{array}{ccc} & & f_{2}≃\exp\left[\frac{A_{12}^{2}{\left(A_{2}^{-1}\right)}_{22}{ß}_{3,c,1}^{2}}{2K_{B}T}\right]{\left[\int_{0}^{2\pi }d\varphi_{2}\int_{0}^{\pi }d\theta_{2}\sin\theta_{2}\right]}^{-1}\int_{0}^{2\pi }d\varphi_{2}\int_{0}^{\pi }d\theta_{2}\sin\theta_{2}\times \\ & & \exp[-\frac{A_{12}^{2}{\left(A_{2}^{-1}\right)}_{22}{\left(u_{2}{ß}_{3,c,1}\right)}^{2}}{2K_{B}T}] \end{array}$$

$A_{12}$ and ${\left(A_{2}^{-1}\right)}_{22}$ account, respectively, for the influences of unit 2 and of all units 2,…,$N_{te}-1$ on the dynamics of unit 1. At this stage, one approximates: $-\left(K_{B}T\right)\left(\nabla_{{ß}_{3,c,1}}\ln f_{2}\right)≃A_{12}\sigma {ß}_{3,c,1}$, $\sigma =-A_{12}{\left(A_{2}^{-1}\right)}_{22}\sigma_{1}$ with constant $\sigma_{1}$, $0<\sigma_{1}<1$.
